# Combined eye tracking and electroencephalography during referential selection in dyadic interaction

**DOI:** 10.3758/s13428-026-03172-0

**Published:** 2026-10-19

**Authors:** Ingmar Brilmayer, Philip Georgis, Petra B. Schumacher

**Affiliations:** University of Cologne, Institute for German Language and Literature I, Linguistics, Albertus-Magnus-Platz, 50923 Cologne, Germany

**Keywords:** EEG, Eye tracking, Naturalistic interaction, Deconvolution, Language

## Abstract

Understanding language in real-world interaction requires methods that integrate auditory, visual, and neural signals. We present a proof-of-concept study combining mobile electroencephalography (EEG) and eye tracking to investigate referential selection during dyadic communication. Using a simplified version of the director task, pairs of participants engaged in naturalistic object-movement instructions while EEG and gaze data were recorded and synchronized via LabStreamingLayer, allowing us to calculate overlap-corrected, regression-based event-related potentials (rERPs) time-locked to spoken nouns and to participants’ fixations, focusing on the N400/P300 (300–500 ms) and later (500–800 ms) time windows. Our results show that gaze behavior and fixation-related potentials provide crucial information for interpreting language-related ERPs: Targets competing with occluded referents elicited a stronger posterior positivity consistent with greater attentional demands. Fixation timing systematically modulated neural responses. Contrary to predictions, competitors received little visual attention, indicating that participants prioritized task-relevant information. These findings highlight the potential of multimodal data integration for understanding attentional and predictive mechanisms in real-world communication. Importantly, we show that established analytic techniques, such as artifact subspace reconstruction, independent component analysis, and linear deconvolution for ERP calculation, apply to noisy, naturalistic datasets. In addition, we provide a subsampling sensitivity approach to test the stability of the effects across different sample sizes. This study provides a methodological framework linking gaze and neural activity during interactive language processing. All preprocessing and analysis scripts, along with example datasets, are openly available on OSF (https://osf.io/5ds4z; a modified and optimized Python implementation is also available on GitHub: https://github.com/XlinCLab/DGAME).

## Introduction: Multimodal language

Language in communication is a complex system of multimodal signals, in which the entirety of a person’s body – from speech and breathing to facial expressions, gestures, and other bodily movements – can be a potential source of information (Goodwin, 2003; Mondada, 2016). In recent years, there has been a notable trend in linguistics to acknowledge this complexity by moving towards a multimodal view of human language (Benetti et al., 2023; Hasson et al., 2018; Holler & Levinson, 2019; Levinson & Holler, 2014; Mazzini et al., 2023; Mondada, 2016; Perniss, 2018; Vigliocco et al., 2014). From this perspective, language in face-to-face interaction is anchored in the immediate environment in which it occurs. Beyond speech, listeners and speakers use information from the visual domain, for instance to establish reference (e.g., Debreslioska et al., 2013; Graham & Argyle, 1975; Hanna & Brennan, 2007; Kita & Özyürek, 2003; So et al., 2009; Somashekarappa et al., 2020).

Most studies of language in real-world interaction have targeted the time-frequency domain, for instance in studies of entrainment, cross-frequency coupling, or inter-brain synchrony (Dikker et al., 2021; Drijvers & Holler, 2022; Pérez et al., 2017). Linguistic event-related potential studies on language in interaction have so far been limited to virtual reality (Huizeling et al., 2023; Tromp et al., 2018; Zappa et al., 2019), probably due to the wide range of difficulties connected with real-world EEG studies, from technical setup, experimental control, and bad data quality, to labor-intensive annotation and complex analyses. For instance, if participants are allowed to speak freely, it can be difficult to identify enough occurrences of comparable linguistic events across participants, making an event-related potential study challenging.

Still, a growing body of evidence from controlled studies suggests that linguistic (i.e., speech) information and cues from the visual channel (e.g., gestures) are simultaneously and immediately processed and integrated by the human brain (e.g., Biau et al., 2016; Hubbard et al., 2009; Özyürek, 2021; Peeters et al., 2017; van Wassenhove et al., 2005; Willems et al., 2008; Wu & Coulson, 2005; Zhang et al., 2021; while most of these studies investigated language comprehension, some have also examined multimodal integration during language production, e.g., Peeters et al., 2017). Using functional magnetic resonance imaging (fMRI), Hubbard et al. (2009), for instance, showed that gestures modulate activity in the auditory cortex related to auditory language processing, providing evidence for the tight coupling of speech and gesture in the brain. ERP studies have repeatedly shown that the N400 and P300 are especially sensitive to multimodal integration (e.g., Özyürek et al., 2007; Peeters et al., 2015; Willems et al., 2008; Zhang et al., 2021). In two EEG studies, Zhang et al. (2021), for example, showed that multimodal cues modulate the N400 event-related potential (ERP) component, usually observable when a word's meaning does not fit its context in the broadest sense (see Kutas & Federmeier, 2011). Zhang and colleagues found interactions in the N400 time window (300–500 ms after word onset) between prosodic accentuation and several types of gestural and facial cues. Other studies report N400 effects in the context of integrating written and spoken language with visual input (e.g., Hirschfeld et al., 2011; Knoeferle et al., 2011; Liu et al., 2011; Sitnikova et al., 2008; Staudte et al., 2021). That is, the probabilistic and predictive integration of incoming linguistic meaning, as reflected in the N400 component (Bornkessel-Schlesewsky & Schlesewsky, 2019), extends beyond the auditory speech signal to include visual cues that are weighted differently depending on their contexts of use (Zhang et al., 2021), indicating the importance of domain general, attention related mechanisms involved in the integration of visual and auditory cues during language comprehension.

In the present manuscript, we demonstrate that it is possible to record and analyze multimodal EEG data (visual, auditory) acquired during real-world interaction and offer an approach of how to integrate gaze and EEG data during data analysis and interpretation. Using time-resolved regression (Dimigen & Ehinger, 2021; Smith & Kutas, 2015a, 2015b), we calculated language- and fixation-related event-related potentials from 128-channel EEG data recorded with mobile recording devices. Because we used mobile recording equipment, participants sat naturally, allowing free movement of the head and upper body. Focusing on a traditional time window in neurolinguistic research, the N400/P300 time-window (300–500 ms after word onset), and a second, later time window (500–800 ms; see section 2 for motivation), we show how gaze data and fixation-related potentials can be informative about language-related potentials, and how the interpretation of the latter can benefit from the analysis of the former. For this purpose, we report behavioral (eye gaze, fixations) and event-related potential (ERP) results from a real-world study using a simplified version of the *director task* (Keysar et al., 2000*)* as a proof-of-concept. Before we elaborate on the present study, we want to discuss three main difficulties related to recording and analyzing multimodal data from real-world environments, and how they can be overcome to benefit from multimodal data recordings.

### Multimodal data recording in real-world environments: Challenges

Recording data from multiple modalities, such as EEG, eye tracking, and audio, while offering insights into the interaction of processes from different modalities (Hasson et al., 2018; Holler & Levinson, 2019; Perniss, 2018), introduces significant challenges in data acquisition, synchronization, and analysis (Gregori et al., 2023). Here, we want to briefly discuss three of these challenges and present solutions to each of them: data synchronization, noisy EEG data and overlapping event responses.

First, each modality has its own temporal resolution. For instance, EEG systems often sample at high rates (e.g., 500–1000 Hz) to capture rapid fluctuations in neural activity; eye trackers usually operate from 120 Hz to over 1000 Hz depending on the device, while audio recordings usually use high sampling rates (44.1 kHz or more) to capture the fine structure of acoustic signals. This mismatch can make it difficult to align data streams for meaningful analysis. Second, recording EEG data from participants who are allowed to speak and move their head and upper body introduces strong non-neural artifacts related to these movements (muscle artifacts and moving recording devices; cf. Gorjan et al., 2022). Third, in naturalistic settings, participants perform many tasks besides processing experimental stimuli; i.e., they process visual and auditory information, plan and produce linguistic utterances, and make fixations, saccades, and other movements. This creates an overlap of the event-related responses of these different events (cf. Dimigen & Ehinger, 2021; Ehinger & Dimigen, 2019; Smith & Kutas, 2015a, 2015b). While traditional laboratory experiments can control this effectively, real-world experiments must account for it during analysis. Below, we discuss our approach to address these three issues.

#### Data synchronization: LabStreamingLayer

Synchronization protocols can tackle varying temporal resolution across data sources. For instance, LabStreamingLayer (LSL, Kothe et al., 2024) is a software framework and protocol designed for real-time data streaming and synchronization in research and experimental environments. LSL facilitates the seamless communication and integration of various data sources and devices over LAN or WiFi. The LSL framework includes libraries and tools for different programming languages (e.g., C++, Python, MATLAB), allowing researchers to create applications that generate, transmit, or receive data streams. The protocol ensures synchronized data streams, which is crucial for accurate analysis and interpretation.

LSL assigns each data sample generated by a source (e.g., a sensor or device) a unique timestamp that reflects the moment of its creation. This timestamp is recorded in the metadata accompanying the data sample. LSL uses a clock synchronization protocol to ensure data-sample timestamps align across different devices and sources. This involves maintaining a shared understanding of time across all involved components; i.e., in a network of data sources, one clock is designated as the “master” clock (in our case, the recording computer). This clock serves as the synchronization reference. Other clocks (in our case, the clocks of the recording devices, i.e., audio, video, EEG, ET), adjust their timing to match the master clock by periodically requesting synchronization updates from the master clock. This provides the necessary adjustments to bring the clocks into alignment. These adjustments are then applied to the timestamps of data samples generated by recording devices, enabling precise analysis of simultaneously recorded data streams over time.

#### Noisy EEG data: ASR and ICA

In naturalistic research settings, EEG recordings are subject to a variety of artifacts from muscle activity, movement, and environmental noise, making it challenging to extract clean neural signals (Gorjan et al., 2022). Our approach uses a combination of independent component analysis (ICA) and artifact subspace reconstruction (ASR) (Delorme & Makeig, 2004; Mullen et al., 2015). ICA decomposes the complex EEG signal into a set of statistically independent components, allowing the isolation and removal of those components that represent stationary, non-neural artifacts (Delorme & Makeig, 2004). ASR, by contrast, is well suited to detect and correct transient, high-amplitude disturbances in the data. It works by reconstructing the underlying brain activity from a subspace defined by the cleaner segments of the recording. Together, ICA and ASR form a powerful, complementary framework that can effectively clean noisy EEG data, even when collected in uncontrolled, real-world environments (see Gramann, 2024 for a very recent review on mobile EEG data).

#### Event overlap

Accounting for the overlap of event-related responses in EEG data (and other time series data) can be achieved using (non-)linear deconvolution (e.g., Dimigen & Ehinger, 2021; Smith & Kutas, 2015a, 2015b). In Smith and Kutas (2015a/b), the deconvolution technique within the regression-based event-related potentials (rERP) framework is introduced as a solution to overlapping event-related potentials (ERPs) in continuous EEG recordings. Traditional ERP methods average epochs time-locked to events, assuming either that responses do not overlap or that they control for overlap via stimulus or experimental design. Yet, in real-world experimental paradigms, events occur in rapid succession, and their corresponding neural responses mix, very likely distorting the observed ERP waveform of interest. The deconvolution approach treats the continuous EEG signal as a (linear) combination of overlapping responses. By formulating a general linear model (GLM), each event is represented by a predictor that spans a defined time window, effectively “tagging” the EEG data with the timing and expected shape of the ERP. The design matrix constructed from these predictors allows for the simultaneous estimation of regression coefficients for each event type. These coefficients can then be directly interpreted or be used to reconstruct the original, overlap-corrected ERPs.

The regression-based deconvolution method (rERP) thus disentangles the overlapping neural responses, enabling more accurate estimation of the true underlying ERP waveform for an event of interest. It also permits the inclusion of additional covariates and interactions, enhancing the capacity to model complex, real-world data where events are not neatly separated into categories. The technique has been implemented in several statistical software environments (e.g., MATLAB and Julia: Ehinger & Dimigen, 2019; Python: Sassenhagen, 2019), providing researchers with tools to analyze continuous EEG data under more naturalistic conditions where event overlap is unavoidable.

#### Benefits: Gaze and fixation-related potentials as cues to language processing

The present study should be understood as a proof-of-concept. We aim to demonstrate that considering the aspects described above allows us to tap into the neural integration of spoken language with visual information during real-world interaction. For instance, when participants listen to an utterance, such as “Move the candle to the left, please” while viewing a real-world visual scene, eye-tracking data can inform us whether or not their gaze is directed toward the target object (candle) at the moment the word ‘candle’ is uttered. This allows us to investigate how visual attention or, more generally, visual information modulates neural responses to language in real time. Furthermore, we can relate fixation-related potentials to language-related potentials within and across experimental variables. For instance, fixation-related potentials show an attentional gain (reflected in a positive component) for looks to target objects, as compared to distractor objects (e.g., Brouwer et al., 2013; Kamienkowski et al., 2012). In the case of referential ambiguities, as in the present study, fixation-related potentials can thus be informative cues as to whether participants treated an object as a target or ‘just’ as a referential, but task-irrelevant competitor. Moreover, studies on natural reading demonstrated that semantically meaningful parafoveal preview can reduce the N400 component when fixating a word during reading (Antúnez et al., 2022; Degno & Liversedge, 2020; Dimigen et al., 2012; Li et al., 2024; Niefind & Dimigen, 2016), revealing that tracking the eyes can also provide important information about the availability of predictive cues during language comprehension. Likewise, studies that use a combination of visual stimuli and spoken or written language, such as the visual world paradigm or sentence-picture verification tasks (Hirschfeld et al., 2011; Knoeferle et al., 2011; Liu et al., 2011; Sitnikova et al., 2008; Staudte et al., 2021), suggest that the degree of similarity between visual and linguistic input is strongly reflected in the N400 time range. Interestingly, not only do visual cues influence linguistic processing, but also linguistic cues can shape visual attention and object processing, as shown in cross-modal language–vision paradigms (e.g., Hirschfeld, Zwitserlood, & Dobel, 2011; Andersson, Ferreira, & Henderson, 2011). More broadly, scene semantics itself (i.e., meaning-based object-context relations) has been shown to guide visual attention beyond low-level salience (Henderson & Hayes, 2017). In the present study, in which participants engage with complex visual scenes while processing spoken language, combined EEG and eye-tracking recordings can thus inform us about how the brain’s electrophysiological responses in the N400 time range are shaped by the (un-)availability of visual and auditory linguistic cues that can be used for predicting upcoming sensory input.

## The present study

### The task

We designed a simplified real-world version of the *director task* (Keysar et al., 2000), in which two participants are seated on opposite sides of a vertical array (shelf) in which objects are placed, for instance, two boxes and two candles. One participant (the director) gives instructions to the other participant (the matcher) to move objects in the shelf. However, some of the objects are hidden from the view of the director. For instance, when one of the two candles is hidden from the director’s view (i.e., it is in the matcher’s privileged ground) and the director asks the matcher to move the candle, a referential conflict arises: from the perspective of the director, the candle is uniquely identifiable, but from the perspective of the matcher, the referential expression is ambiguous, unless the matcher considers the perspective of the director. The present study analyzes participants in the role of the matcher, i.e., from a comprehension perspective. The director’s productions, gestures, and gaze are not the focus of the present analyses.

We chose this task for several reasons. First, it is well suited to examining how gaze behavior and language-related event-related potentials integrate during referent selection in a naturalistic experimental setting, since each instruction must be followed by visual identification of the target object before it can be moved. Second, the director task can be designed to be repetitive enough to be used in an event-related potential study, which usually requires a large number of comparable trials to produce reliable results. Finally, there is a rich literature on gaze behavior in the director’s task (e.g., Barr, 2008; Epley, Keysar, et al., 2004; Epley, Morewedge, et al., 2004a, 2004b; Ferguson & Breheny, 2012; Hanna et al., 2003; Heller et al., 2008; J. J. Wang et al., 2019), and there also exist two ERP studies (Richter et al., 2020; Sikos et al., 2019) that used computerized versions of the director task to which we can relate our results.

In the years following the first eye tracking study of the director task (Keysar et al., 2000), the literature was dominated by a debate about the time point in processing at which participants consider the perspective of others, early (Heller et al., 2008; Hanna et al., 2003; Ferguson & Breheny, 2012; Epley et al., 2004b) or late (Keysar et al., 2000; Barr, 2008; Wang et al., 2019; Barr, 2016). Today, it is generally accepted that whether and at which time in processing participants consider the perspective of others depends on a variety of factors, including (linguistic) context (e.g., Heller et al., 2008; Brown-Schmidt et al., 2008), task (Ferguson et al., 2015; Ryskin et al., 2020), culture (Wu & Keysar, 2007; Wu et al., 2013; Wang et al., 2019), and the availability of cognitive resources (Epley et al., 2004a; Cane et al., 2017). Hanna et al. (2003), for instance, showed in two experiments that addressees cannot ignore visually salient objects in privileged ground if they match a speaker's description and assign reference as early as possible once a potential referent is identifiable. Recently, a probabilistic model of reference resolution has been developed assuming that the knowledge of the self and the (assumed) knowledge of the other in dyadic interaction are assigned a weight and are then combined into a probabilistic communicative model by both speaker and addressee (Mozuraitis et al., 2018; Ryskin et al., 2020; Heller & Brown-Schmidt, 2023). This approach highlights that the other’s knowledge can only be *inferred* based on weighted cues from different available sources. In the director task, one of the rather salient and informative cues for the director’s knowledge and intended reference is visual: whether a potential referent of a referential expression is occluded or not. Looks to privileged ground competitors thus occur because they are informative when it comes to inferring the knowledge of the director – participants are collecting evidence for or against a referential choice. On the speaker’s side, complementary work using referential communication tasks has shown that common ground influences language production already during early speech planning (Vanlangendonck, Willems, Menenti, & Hagoort, 2016), specifically when speakers have to distinguish common from privileged ground in communicative contexts (Vanlangendonck, Willems, & Hagoort, 2018). The present study focuses on the comprehension side of this kind of referential conflict.

To the best of our knowledge, there exist only two event-related potential studies using versions of the director task. The first study by Sikos et al. (2019) utilized temporary referential ambiguities and the Nref ERP component, a sustained negativity interpreted as a marker of referential ambiguity (Van Berkum et al., 2007). On a computer screen, participants saw displays of cards showing animals with accessories, e.g., a brontosaurus with boots and a brontosaurus with a purse, such that when participants were asked to “move the brontosaurus with boots”, “brontosaurus” was referentially ambiguous until the word “boots”. They found a graded Nref effect that has the highest amplitude when the referential competitor is in common ground, followed by trials with no competitor and trials with a competitor in privileged ground. They argue that the reduced Nref in trials with privileged-ground competitors shows that participants do not consider privileged-ground competitors as potential referents in the same way they do common-ground competitors. In other words, they consider the other's perspective when determining referential candidates. In the ERP study by Richter et al. (2020), participants were presented with 4 × 4 grids of objects on a computer screen. Referential ambiguities were created using size-contrasted object triplets (e.g., small, medium, large candle) of which either the large or small object was in privileged ground (hidden from the director). When the director now asks to “move the small candle” while the small candle is in privileged ground of the addressee, a referential ambiguity arises: the small candle in privileged ground and the medium candle in common ground are potential referents of the utterance from the listener’s perspective. In two separate studies, Richter et al. (2020) recorded eye tracking and EEG data using the same experiment, creating comparable data sets of two modalities. While the behavioral results and the results of the eye-tracking study replicate previous findings (longer reaction times and an increased number of looks to privileged ground competitors in *pair* trials), the ERP results are different from Sikos et al. (2019). Across conditions, Richter et al. (2020) found a broadly distributed, positive ERP component with posterior maximum that was more pronounced (more positive) for trials with privileged ground competitor as compared to trials without, which is the opposite of the negative effect in Sikos et al. (2019). Richter et al. (2020) interpret this late positive effect as reflecting difficulties associated with the integration of privileged ground information.

**Fig. 1:**
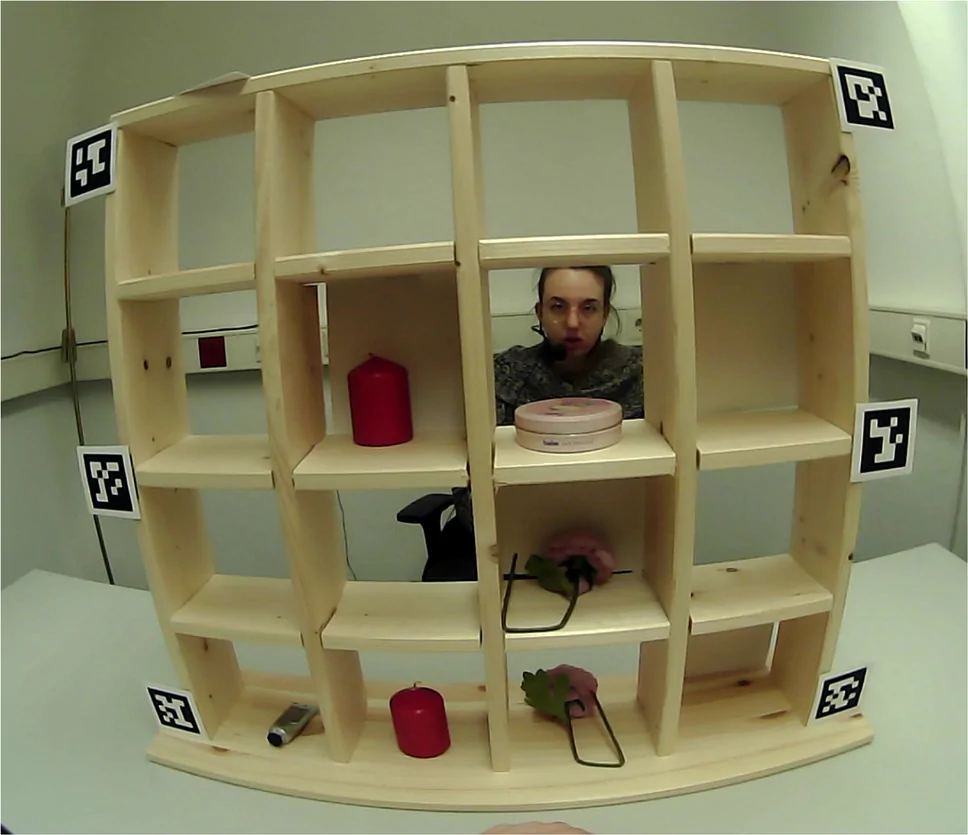
Experimental setup in the current study

In the present study, we used a version of the director task modified in order to make it very simple for participants and to reduce the amount of variance in our data. We used a 4 × 4 shelf on a table as visual display and placed six objects on it (Fig. 1). While there were two singleton, i.e., unique objects (e.g., one cream jar, one tube), four objects were pairs of identical objects (e.g., two candles, two roses), with one object from each pair hidden from the director’s view. We chose this very simple design because the matcher would only need one piece of information to resolve the referential ambiguity: Is an object occluded from the director’s view, or not? In so doing, we aimed to minimize eye movements related to the comparison of objects for decision making: In Richter et al.’s (2020) study, for instance, participants had to compare sizes of objects to resolve the referential ambiguity, in Sikos et al.’s (2019) study, participants had to compare accessories (e.g., a Brontosaurus with boots and one with a purse), thus possibly fostering looks to the competitor object, in order to collect evidence for or against a referential choice. Interestingly, no comparison was necessary. Participants could either look at the competitor, see that it is hidden and then switch to the target, or they could look at the target, see that it is not hidden, and move it. Effectively, this makes the target the most informative object in the present study, because it provides the necessary visual information (hidden or not) *and* because it is the actual target object. Accordingly, we expected participants to pay little attention to the hidden competitors, signaling no difficulties in taking the perspective of the other into consideration. Since we recorded EEG and gaze data of the matcher, we are able to directly test this assumption by tracking where participants’ gaze was directed while they heard a target word and by considering it as a predictor in our rERP calculation. Likewise, we included this information in the calculation of the fixation-related potentials in order to examine differences between trials in which participants were hearing or had already heard the target word while fixating the target object, and trials in which they were not. Here, we focus on the N400/P300 time range (300–500 ms) and include an additional later time window (500–800 ms) to account for later effects that we expected based on the very long effect latencies in Richter et al. (2020) and Sikos et al. (2019). We assume that if participants collect sufficient visual information about the target object (e.g., its position on the shelf) before they have linguistic evidence for the identity of the upcoming target (mean target fixation time before noun, i.e., negative value), this possibly facilitates target selection during and after linguistic input (e.g., noun onset) in a top-down manner. We assume that this (predictive) information is reflected in a reduction in N400 amplitude and/or an increase in P300 amplitude. In trials in which participants made the majority of target looks during the noun (*during noun;* mean target fixation 0–471 ms), visual target information and linguistic target information processing occur simultaneously, with visual and auditory linguistic input providing bottom-up information at the same time. The relative absence of a priori visual information about the target object (e.g., its position) probably leads to relative difficulties in target detection, as compared to the *before noun* cases. This should be reflected in a negative ERP effect between 300 and 500 ms, e.g., an increase in the centrally distributed N400, and a decrease in the occipital P300. Finally, in trials in which the mean of all target looks occurs later than the offset of the critical noun, participants already have complete linguistic evidence regarding the identity of the target stimulus before they start actively searching for the object on the shelf. That is, it mirrors the *before noun* cases, where visual information is available before linguistic information, which is the opposite in the *after* noun cases. Again, we expect this to be reflected in a further decrease in P300 amplitude (difficulties in target selection and task execution) and an increase in the N400 amplitude (integration difficulties since no target has been visually identified).

Before we turn to the Methods section, we would like to make the scope of the present analyses explicit. As outlined above, the present study focuses on the matcher’s gaze and EEG during referent selection. The director’s productions, gestures, gaze, and facial expressions are not part of the present analyses. This is a deliberate methodological decision: we aimed to demonstrate the feasibility of the deconvolution-based multimodal approach on a single, clearly defined data stream from one of the two interlocutors before extending it to the full multimodal recording from both.

## Methods

### Participants

Twenty-six native speakers of German (14 women, mean age 24.94, range 20–32) were analyzed for the current study. We excluded an additional seven participants from the analysis due to technical problems. Of these, four exclusions resulted from an eye-tracking glasses cable that was mistakenly unplugged, two were due to human error, and one was due to a loss of WiFi connectivity. Participants provided written informed consent before the recording session and received either course credits or monetary compensation for their participation. Recording sessions lasted between 2 and 3 h per dyad, including setup, calibration of the mobile eye tracker, and EEG impedance check, and required trained confederates throughout.

The sample size was determined by feasibility considerations rather than by an a priori power analysis. To our knowledge, no closed-form *a priori* power analysis is currently available for the deconvolution-based regression-ERP framework with permutation-based second-order statistics, which we use here. Our sample size is in line with comparable naturalistic dyadic-EEG studies (Kawasaki et al., 2013: *N* = 19; Pérez et al., 2017: *N* = 15; Pérez et al., 2019: *N* = 30; Drijvers & Holler, 2022: *N* = 31; Mazzini et al., 2025: *N* = 24; Yamasaki, Blache and Schön, 2026: *N* = 18). We address this issue more thoroughly below: we performed a subsampling sensitivity analysis and discuss two complementary simulation-based power analysis frameworks that could also be applied within the current framework.

### Experimental stimuli and procedure

In the experiment, a participant and a confederate were seated face-to-face on opposite sides of a table. The matcher’s task was to move objects around a 4 × 4 shelf placed in the middle of the table according to the instructions of the director. Four of the 16 compartments of the shelf were occluded from the view of the director. Six objects were placed on the shelf: two were singleton items (*singleton)*, and the other four were two pairs of identical objects (*pair)*. In two of the hidden compartments, one object from each pair of identical objects was placed. The other two hidden compartments remained empty. In German, gender is marked on the determiner preceding a noun and can be used as a cue for referential predictions when a set of potential referents with different grammatical gender exists. In order to avoid such predictions, all objects used in the current experiment represented bisyllabic feminine names (*singleton objects*: *die Pflanze*, ‘plant’; *die Spritze*, ‘syringe’; *die Flasche*, ‘bottle’; *die Dose*, ‘can/tin’; *object pairs*: *die Kerze*, ‘candle’; *die Blume*, ‘flower’; *die Vase*, ‘vase’; *die Tasse*, ‘cup’). The objects were grouped into two sets of two critical pairs and two singleton objects. The grouping of objects into sets was counterbalanced across participants. In the experiment, all participants saw all objects across four experimental blocks. In Block 1 and Block 2, the objects from set A were used; in Block 3 and Block 4 the objects from set B. We counterbalanced the order of object sets and shelf-arrangement patterns across participant pairs using a Latin-square design. During the experimental pauses after Block 1 and Block 3, our lab assistants rearranged the objects on the shelf and in the hidden compartments ad hoc to respect the design constraints (one object of each pair occluded; singletons never occluded). The lab assistants were aware of the experimental design but not involved in formulating hypotheses about gaze behavior or ERP effects, and they had no access to the recorded data during the session. The directors were instructed to move every object on the shelf 10 times within one experimental block, record the target position on the shelf, avoid moving the same object twice in a row, and use ‘left’ and ‘right’ from the matcher's perspective. Matchers were instructed to move objects only one compartment at a time in any direction and to keep in mind that they could see more than the director.

The experimental procedure was very simple. After setup or reconfiguration, the lab assistants left the room, and the director started with the instructions. Each block ended after every object had been moved ten times. The experiment lasted between 35 and 60 min. Including setup, each session lasted between 2 and 3 h.

### Data recording

All data were recorded and synchronized with the other data streams via labstreaminglayer (LSL; https://github.com/sccn/labstreaminglayer) using the LabRecorder app for LSL (https://github.com/labstreaminglayer/App-LabRecorder).

#### Audio data

We recorded the voice of the confederate using a headset microphone (AKG C 520). The recording was digitized with a sampling rate of 22050 Hz (Mono) using an external audio interface (Focusrite Scarlett 2i2 3rd Gen).

#### Eye tracking data

We recorded participants’ gaze using Pupil Core eye tracking glasses (Pupil Labs) with a monocular setup. The Pupil Core headset records pupil data at a 120-Hz nominal sampling rate and a second (“world”) camera records the scene from a first-person perspective with a sampling rate of 30 Hz. We used single marker calibration as implemented in the recording software PupilCapture. Angular accuracy and precision were kept below 1.5° and 0.15°, respectively. We recorded the data to a hard drive and synchronized it with the other data streams via LSL. To track the positions of the objects on the shelf throughout the recording, we attached four AprilTags (Olson, 2011; Krogius et al., 2019; Wang & Olson, 2016), which function similarly to QR codes, to the shelf, one in each corner. Using the surface tracking plugin provided by Pupil Labs, we defined surfaces (areas of interest) offline (here, the 16 compartments of the 4 × 4 shelf). An algorithm then calculates the distances between the tags and the surfaces for every video frame, allowing us to correct for viewpoint changes (e.g., head or body movements) and identify each surface's location throughout a recording. Thus, we can track which of the 16 surfaces a participant is looking at at any given time.

#### EEG data

EEG Data were recorded using a CGX-mobile-128 System (Cognionics, San Diego, CA, USA) with 128 AgCl active electrodes relative to a left mastoid reference at a sampling rate of 500 Hz. The right mastoid served as ground. Four of the 128 channels were never recorded (‘FTT10h’, ‘FTT9h’, ‘FFT10h’, ‘FFT9h’) because they were removed to fit the eye-tracking glasses. Channel impedance was kept below 20 kΩ at the beginning of the experiment, which is adequate for a setup using active electrodes.

The EEG was digitized and synchronized with the other streams via LSL using the LSL-relay implemented in the CGX-Recording software. We recorded each experimental block to a separate file (2 patterns and 2 object sets = 4 files), since between blocks participants moved freely and did not perform any task-related actions. We also used this as a security measure so that, in case of data loss (e.g., due to connectivity issues), we would not lose an entire experimental session.

### Data preprocessing

#### Code availability

The scripts that were used for the analyses reported below can be found online on OSF (https://osf.io/5ds4z/files/osfstorage, matlab/R). We also have created a version of the scripts with much lower runtime and optimized code that is actively maintained and developed further (https://github.com/XlinCLab/DGAME).

#### Audio data and annotations

The recorded audio data in xdf format were imported into MATLAB, where it was normalized and exported to .wav. Automatic speech recognition and automatic segmentation were then performed on the resulting audio files (Schiel, 1999, 2015) via the BAS web interface (Kisler et al., 2017). Afterward, we manually reviewed the output and applied corrections as needed. Manual corrections mostly involved misplaced fricative onsets and unclear pronunciations. We then manually annotated the audio recordings for experimental conditions. During the experiment, we also instructed the directors to write down the current positions of the objects on the shelf. This information was then manually transferred into lists and combined with the annotations using custom scripts. This enabled us to match the gaze data with the current object positions automatically.

To get an idea of the variation in the linguistic input, we performed a brief descriptive analysis of the linguistic constructions used by the director. Across all instructions, the mean number of words per instruction was 7.64 words. The median length of the critical noun phrase (e.g., *die Kerze,* ‘the candle’) was 641 ms, and the median duration of the critical noun was 446 ms. To characterize the surface form of the directors’ speech, we extracted from the ASR transcripts the sequence of part-of-speech-like tags D (determiner), N (noun) and DIR (directional element, e.g., *links*, *rechts*, *oben*, *unten* [‘left’, ‘right’, ‘up’, ‘down’]*)* and reference to the goal of the movement by *einen* (‘one’*)* together with the connective particles *und* (‘and’) and *dann* (‘then’) and the politeness/discourse markers *bitte* (‘please’). Table 1 lists the ten most frequent surface patterns. The canonical core *D N nach DIR* (D N ‘to’ DIR, e.g., *die Tasse nach oben, ‘the cup up’*) and its connective-extended variants *und/dann D N nach DIR* (‘and/then’ D N ‘to’ DIR) together accounted for 21.6% of all instructions; including *bitte*-marked variants and patterns with multiple DIR specifications (e.g., *nach oben links, lit. ‘to up left’, ‘to the upper left’*), the ten most frequent patterns covered approximately 40.83% of the dataset. The remaining instructions were distributed over a long tail of less frequent surface variants. We did not enter these surface patterns as predictors into the analyses reported below; we provide them here purely as a descriptive characterization of the linguistic input the matchers received.

**Table 1:** Instruction patterns used by the director over the entire experiment with a relative frequency of >1 % of all instructions (= 40.83 % of all instructions). Translations and abbreviations: und, ‘and’; nach, ‘to [the]’; dann, ‘then’; bitte, ‘please’; einen, ‘one’; D: determiner; N: noun; DIR: direction (left, right, up, down and combinations thereof)

Pattern	*N*	%
‍und D N nach DIR	358	8.04
‍dann D N nach DIR	333	7.48
‍D N nach DIR	272	6.11
‍D N bitte nach DIR	256	5.75
‍und D N nach DIR DIR	130	2.92
‍dann D N nach DIR DIR	119	2.67
‍D N bitte einen nach DIR	96	2.16
‍D N nach DIR DIR	91	2.04
‍D N bitte nach DIR DIR DIR	83	1.86
‍dann bitte D N einen nach DIR	80	1.8
‍total	1818	40.83

#### Eye tracking data

The preprocessing of the eye tracking data was carried out in R using the library eyetrackingR (Forbes, Dink & Ferguson, 2021). Any data with a pupil-detection confidence <.60 were excluded from analysis, and non-AOI (area-of-interest) looks were not treated as missing. As there is no pre-existing software or package available that can handle this specific type of data we recorded (e.g., our data is not trial-based but continuous gaze data), much of our data preprocessing pipeline consisted of custom scripts to produce the required data formats for analysis (e.g., combine the gaze data with the linguistic annotations, create trials).

#### EEG data

The EEG recordings of each block were imported into EEGLAB (Delorme & Makeig, 2004) using the MobiLab plugin (Ojeda et al., 2014). Before any preprocessing steps, we created an event structure for each block containing all words spoken by the director and all fixations of a participant. The data were resampled to 250 Hz. Afterward, the data were concatenated and preprocessed as follows. We first filtered the data with a 2-Hz FIR filter (pop_firfiltnew, cutoff frequency (– 6dB): 1 Hz) to approach stationarity for later ICA decomposition. Afterward, we rejected noisy channels semi-automatically. First, we rejected channels selected by visual inspection of the raw EEG data. Then we used the *pop_clean_rawdata()* function to automatically reject noisy channels, followed by automatic rejection using the kurtosis method of the *pop_rejectchan()* function with a threshold of 2. Afterward, we applied a 100-Hz low-pass filter to the data and then used the function *cleanLineNoise* to remove line noise artifacts at 50 Hz. Then, we used artifact subspace reconstruction (ASR; Mullen et al., 2015) as implemented in the *clean_rawdata* plugin for EEGLAB to identify and correct noisy segments in the recording.

After ASR, we interpolated missing channels (mean 26, range 17–46) and re-referenced the data to an average reference under reconstruction of the original reference. After re-referencing, we removed the original reference and interpolated channels again to avoid rank deficiency. Then, we used the AMICA algorithm to decompose the data into independent components (one model, 2000 iterations, ten times rejection of unlikely data). The resulting weights and sphere of the model were then transferred to a 0.3-Hz high-pass and 20-Hz low-pass filtered version of the data with 250-Hz sampling rate. We then used the ICLabel plugin for EEGLAB to classify components and subsequently rejected all components not labeled “brain” (mean 43, range 14–62).

### Statistical analysis

#### Gaze data

We examined three measures of the gaze data: to investigate the time-course of target selection, (i) proportion of looks to the target object and the competing object in a pair (the former being in common ground, the latter in privileged ground); (ii) proportion of looks to competitor relative to all looks to other non-target objects; and (iii) the mean time point at which participants looked at the target object for each trial, which we used as a predictor for the ERP analysis. This measure summarizes all looks to the target, collapsing the first look to the target, the last look to the target, and all looks in between into a single value. While this has the disadvantage of being rather imprecise, as for instance, compared to the first look to a target object, it provides a measure for the processing load associated with target identification throughout an entire trial and beyond the single-word level. This measure is thus comparable to the *total reading time* in eye tracking research on reading (e.g., Kliegl et al., 2004, 2006; Kuperman et al., 2018).

We contrasted the target of a pair (which conflicts with an object in privileged ground; hence “*pair condition*”) with the target of a singleton (“*singleton condition*”). We used a cluster-based permutation analysis (Maris & Oostenveld, 2007) to test for significant differences in the proportion of looks between the *pair* and the *singleton* condition for the target AOI (cf. Richter et al., 2020). We calculated a paired, two-sided *t* test for 100-ms time bins from 0 to 3500 ms after critical noun onset. For all time bins with a significance threshold of 0.05, we then repeated the procedure 4000 times with randomly shuffled (not sequentially ordered), i.e., permuted, versions of the binned data. This produces a distribution that is expected at chance level. We then compare these results to the original model using the sequentially ordered time series. This method yields a *p* value that tells us the likelihood of detecting a given effect when the distribution was random, i.e., if there was no effect of our experimental variable.

Since in the *singleton* condition, the privileged ground competitor was absent, we could not compare looks to the privileged ground competitors between conditions in the same way. Instead, we compared the proportion of looks to the competitor object in the *pair* condition with the proportion of looks averaged over all other, non-target objects: the two singleton objects and the two objects from the other pair (one in privileged ground, one in common ground).

##### Subsampling sensitivity analysis

An open question in the framework we present is how to determine statistical power. Here, we perform a sensitivity analysis on our dataset to assess the reliability of our method with respect to sample size. We performed a subsampling sensitivity analysis (cf. Schönbrodt and Perugini, 2013; Politis, Romano & Wolf, 1999) on the cluster-based permutation tests of the gaze data. For each sample size *N* from 15 to 25, we drew 200 random subsamples without replacement, repeated the cluster permutation procedure described above (4000 permutations, paired two-sided *t* test on 100-ms bins, identical AOI definitions and contrasts) on each subsample, and computed the proportion of subsamples in which the cluster reached significance at α = 0.05. We report this as the significance rate, and the smallest *N* at which the significance rate reached or exceeded 80% as the threshold N, separately for each of the three gaze contrasts (target, competitor, goal). The full code for this analysis is openly available in the project repository, in a standalone Python script that reproduces the original R-based permutation analysis exactly (Pearson correlation between Python and R *t*-statistics: *r* = 1.0; maximum absolute deviation across 1200 values: 8 × 10⁻^12^).

#### EEG data

##### First-order statistical analysis: rERP calculation

In order to account for the overlap between brain responses to the stimuli of interest (critical nouns) and other stimuli (determiners, words following or preceding the noun, fixations), we calculated a general linear regression model with a linear time basis function from – 500 to 1500 ms after event onset for four different event types: determiner preceding the noun, the critical noun, words preceding the determiner (max. 2), intervening between determiner and noun, and following the noun (max. 2), as well as fixations within – 1500 to 3500 ms after noun onset. We were interested in the event-related potentials following critical nouns and fixations; the other events were only included to account for the overlap. For the determiners, we included the experimental condition (*pair, singleton*) as a categorical predictor and added *trial* as a covariate without interaction. For critical nouns, we additionally added a continuous predictor, *mean target gaze time* (see section Gaze data), for which we assumed an interaction with condition. For all other words before the determiner, or before or after the noun, we only calculated an intercept model to account for the overlap. The following list contains the formulae used to compute the overlap-corrected ERPs for all four event types described above.

1. determiner preceding critical noun: µV ~ condition + trial
2. critical noun: µV ~ condition * mean_target_gaze_time + trial
3. preceding, intervening and following words: µV ~ 1
4. fixations within – 1.5 to 3.5 s around critical nouns: µV ~ 5. condition * fixatedObject * trial_time+spl(saccadic Amplitude)

We used the resulting beta estimates to reconstruct the ERPs of critical nouns and fixations, including the conditional means of the covariates (nouns: trial, fixations: saccadic amplitude).

### Second-order statistical analysis: Permutation analysis

For the second-order statistical analysis of the language-related ERPs, we summarized the ERP data into 100 ms time bins to account for autocorrelation. We then calculated a linear regression model for each of the resulting time bins using the following formula:

$$\mathrm{data}\sim\mathrm{laterality}\times\mathrm{sagittality}\times\mathrm{condition}\times\mathrm{mean}\_\mathrm{target}\_\mathrm{fixation}\times\mathrm{baseline}$$

Here, *baseline* (mean predicted microvolt – 250 ms to 0 ms relative to noun onset) was added to the model formula to apply robust baseline correction (Alday, 2019), and sagittality and laterality were categorical topographic predictors (laterality: left, central, right; sagittality: prefrontal, frontal, central, posterior, occipital). We then used a time bin-based permutation approach to test for significant effects. For this purpose, we shuffled the predicted microvolt values within each time bin to decouple the dependent and independent variables, thereby simulating the null hypothesis, under which the predictors of our model and the dependent variable (predicted microvolt) are not related. Then we calculated linear models for 2000 permuted versions of each time bin and compared their absolute t values *|t|* to the original, unshuffled models’ *|t|* values. From this comparison, we created a probability distribution for each time bin, making it possible to compute how likely *|t|* is larger under the assumption that the null hypothesis is true, with smaller *p* indicating lower probability that the null hypothesis is true and higher probability that the effect of the unshuffled data is a true effect.

The statistical procedure for the fixation-related ERPs was identical to the one described for the nouns above. The model formula included the same topographic factors, a factor for *condition* (pair/singleton), a continuous predictor for the *trial_time* relative to noun onset at which a fixation occurred, and a continuous predictor *baseline* (– 250 to 0 ms relative to fixation onset). For the second-order analysis, we only focused on the reconstructed rERPs for target objects, dropping all other fixation-related potentials.

#### Subsampling sensitivity analysis

In parallel to the gaze data, we performed a subsampling sensitivity analysis on the second-order statistics described above (cf. Schönbrodt and Perugini, 2013; Politis, Romano and Wolf, 1999). The aim was to determine the sample size at which the central condition effects in our data become reliably detectable, given the present analytic framework. We emphasize that this is not an observed-power calculation in the sense of Hoenig and Heisey (2001); rather, it is a resampling-based assessment of the stability of significance decisions across reduced sample sizes. For each sample size *N* from 15 to 25, we drew 200 random subsamples without replacement, ran the identical permutation procedure as described above (2000 permutations, robust baseline correction, identical model formulae), and computed the proportion of subsamples in which a given effect reached significance at α = 0.01/0.05/0.1/0.2 in a given time bin. We report at which value of *N* the significance rate reached or exceeded 80%, 90% or 95% (denoted threshold *N*), separately for each effect of interest, time bin, and electrode region of interest (ROI), as well as a global summary across time bins. As for the gaze data, we provide the full code for this analysis in the public project repository.

#### Simulation-based power analysis: outlook

Beyond the subsampling sensitivity analysis described above, simulation-based power analysis for deconvolution-based regression ERPs with permutation-based second-order statistics could also be applied within the current framework. The first approach would simulate the entire analysis pipeline, from raw EEG to second-order statistics, using empirical noise PSDs, spatial covariance, event timing distributions, and effect sizes estimated from the present dataset; this allows estimation of detection rates under varying effect-size scaling but is computationally intensive and is only feasible with high-performance clusters. The second approach operates at the level of the per-subject regression coefficients (betas) and reconstructs ERPs from grand-averaged betas plus per-subject deltas, scaled by an effect-size factor; this removes the deconvolution step entirely and brings computation time down by approximately two orders of magnitude. A full methodological treatment of power estimation in this analytic framework is, however, non-trivial and warrants a publication of its own.

## Results

### Gaze data

**Fig. 2:**
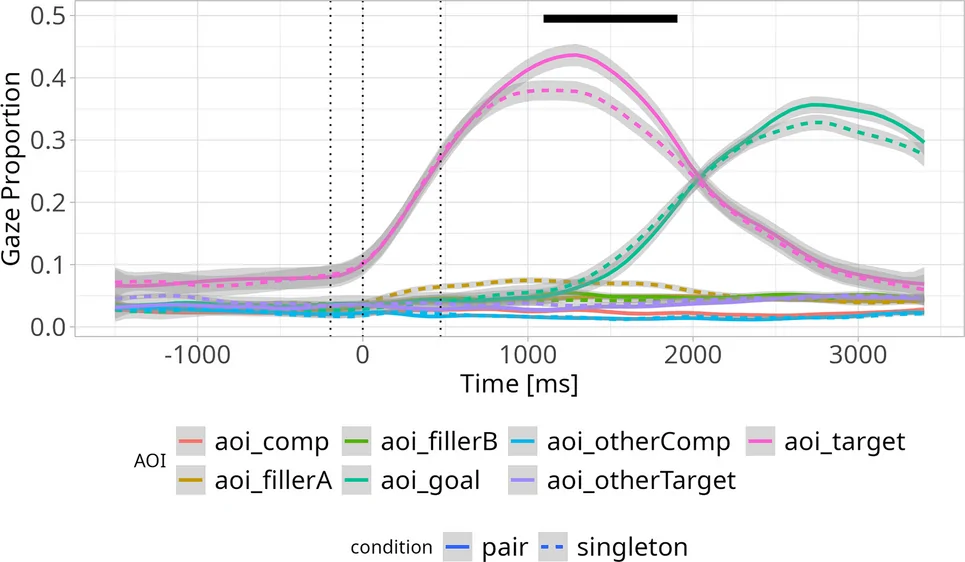
Gaze proportions by condition (solid: pair, dashed: singleton) and AOI (color). The black bar marks significant differences between conditions. Abbreviations: target: the target object; comp: competitor; fillerA/B: singleton, non-target objects; otherComp: the competitor of the non-target pair; otherTarget: the non-hidden target of the other pair; goal: the goal compartment, i.e. the intended end point of the target object in the shelf. Vertical dashed lines from left to right: median determiner onset, noun onset, median noun offset

**Fig. 3:**
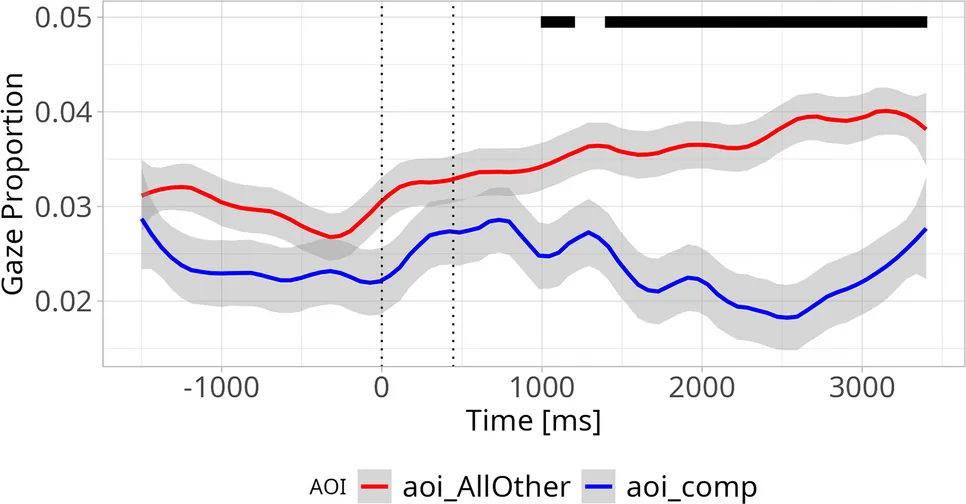
Proportion of looks to the hidden competitor (blue) and the mean over all other objects in the shelf (red) in the pair condition. Numerically, the proportion of looks to the hidden competitor is lower at all time points from −1.5 to 3.5 seconds after noun onset. Statistically significant differences are marked by black bars

Figure 2 shows the proportion of looks to the different objects in the shelf by condition. The figure reveals a higher proportion of looks to the target object in the *pair* (solid) than in the *singleton* (dashed) condition. The cluster-based permutation analysis of the target-AOI revealed a significant effect in a cluster between 1100 and 1900 ms after critical noun onset (*p* = 0.009). In a post hoc analysis of the goal-AOI, although numerically present, the difference between conditions did not reach statistical significance (*p* > 0.1). Since there were no privileged ground competitors in the *singleton* condition, we compared the proportion of looks to privileged ground competitors in the *pair* condition with the mean proportion of looks to all other, non-target objects (Fig. 3): the two singleton objects and the two objects from the other pair (one in privileged ground, one in common ground). As Fig. 3 demonstrates, the proportion of looks to the hidden competitor is numerically lower than the mean proportion of looks to all other non-target objects on the shelf. According to the results of the permutation analysis, this difference is even statistically significant in clusters between 1400 and 3400 ms (*p* = .001) after stimulus onset.

### Language-related ERPs

**Fig. 4:**
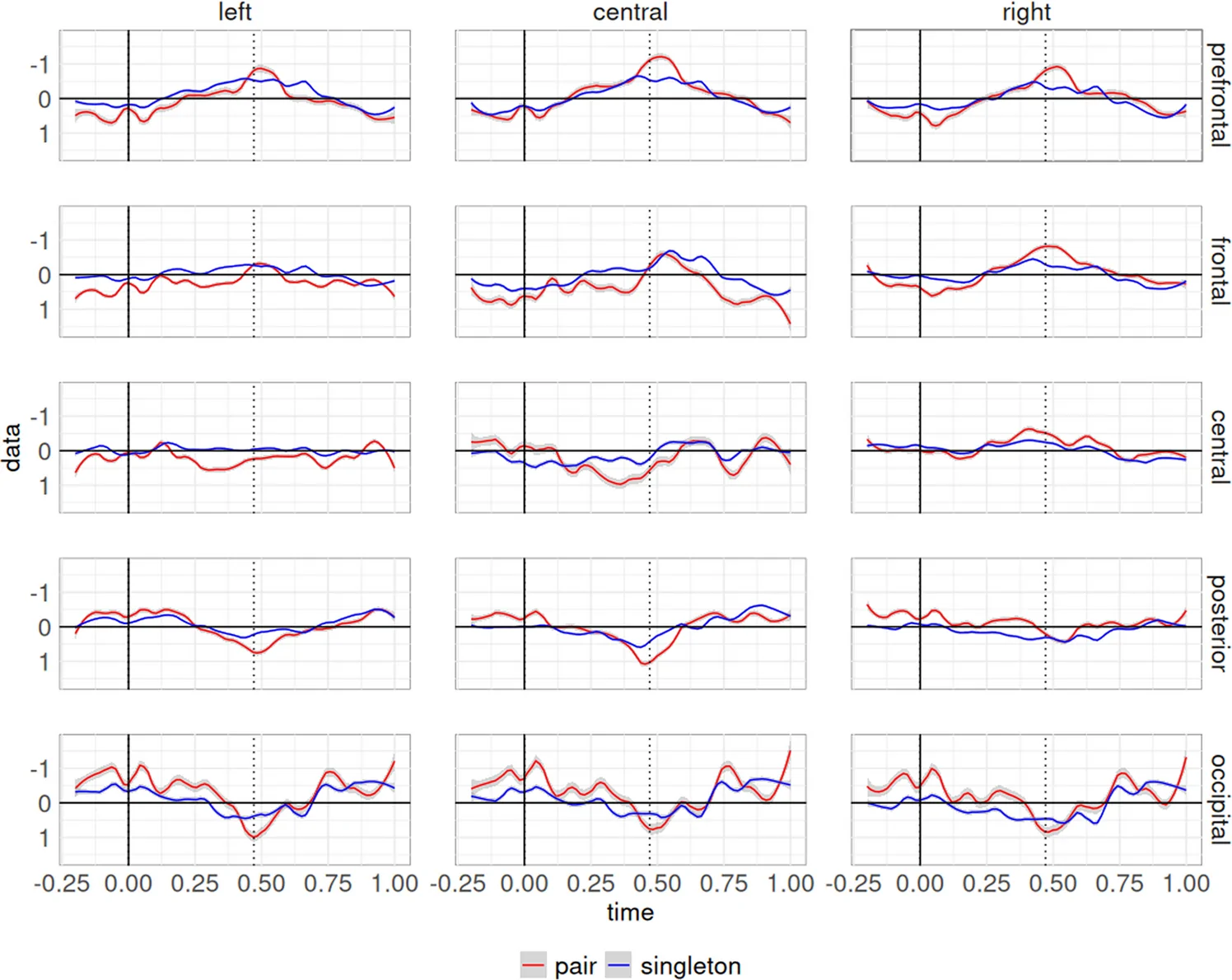
Overlap-corrected, reconstructed ERPs time-locked to noun onset by condition and region-of-interest. The dotted vertical line marks the median noun duration

Figure 4 shows the overlap-corrected, reconstructed ERPs by condition and ROI. These can be interpreted in the same way as traditional grand-averages. The most pronounced patterns include a positivity for the *pair* condition (~ 250–500 ms) spanning left and central prefrontal, frontal, and central ROIs, followed by a later positivity for the *pair* condition with a posterior maximum peaking at ~ 500 ms after stimulus onset. The frontal negativity clearly visible for the *pair* condition at ~ 500 ms over central- and right-prefrontal ROIs that extends to the frontal ROI over right-hemispheric channels is very similar in timing and morphology as compared to the posterior-occipital positivity and probably reflects a common underlying dipole. Moreover, a negativity for the *pair* condition is visible between 0 and ~ 400 ms at left-, right- and central-occipital channels. Over right-hemispheric channels, it is also visible at the posterior and central ROI. Finally, there is a later positivity at left-frontal and centro-frontal ROIs between ~ 600 and 850 ms for the *pair* condition. All other differences between conditions seem rather small and will not be considered.

**Fig. 5:**
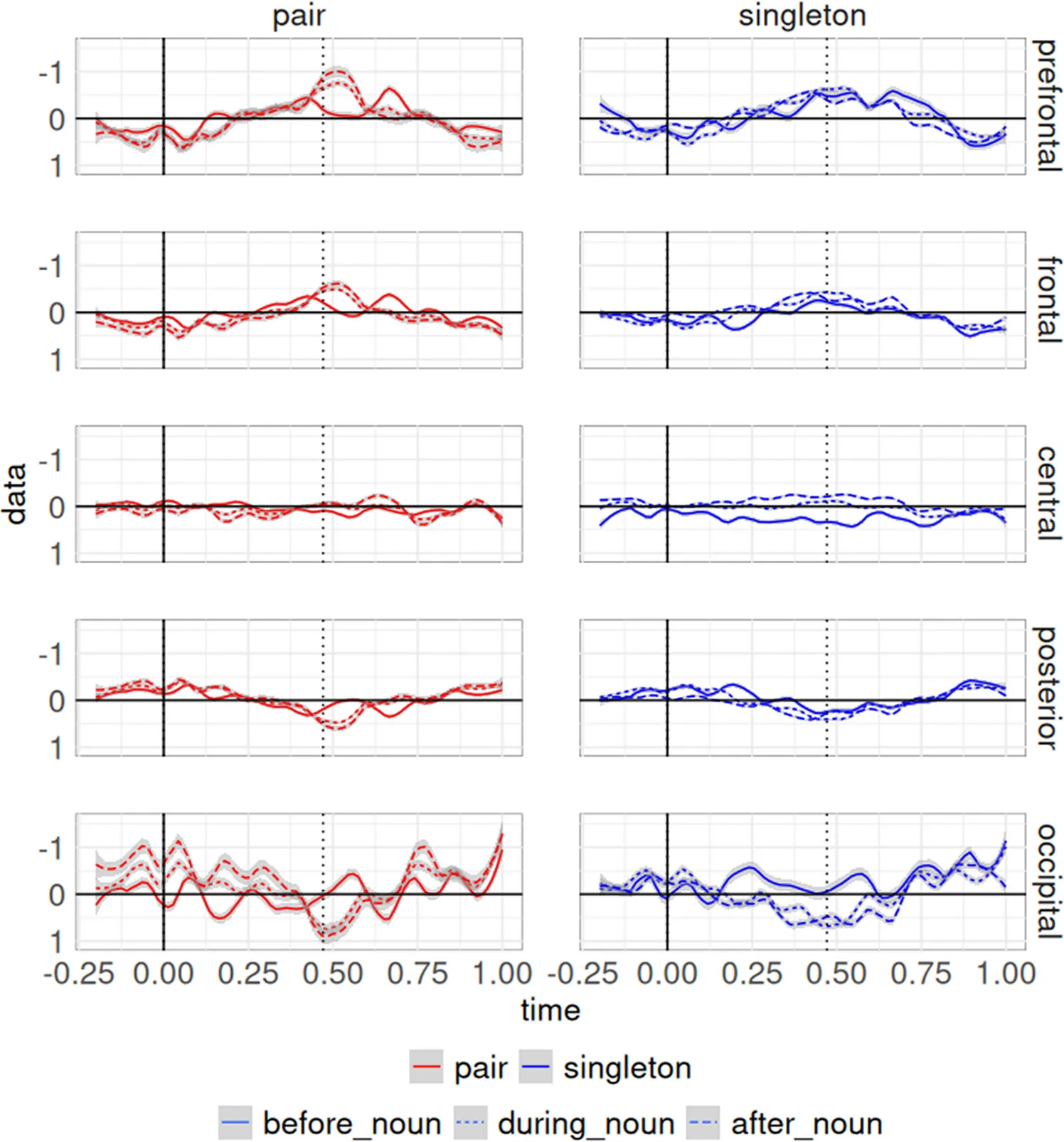
Overlap-corrected, reconstructed ERPs time-locked to noun onset by condition, sagittal regions-of-interest and mean target fixation time. The dotted vertical line marks the median noun duration

Figure 5 shows the ERPs to critical nouns in the *pair* (left) and *singleton* (right) condition, grouped by ROI (sagittal axis only) and the mean time point relative to noun onset at which participants fixated the target object (grouped for plotting purposes only; *before noun*: – 1000ms to 0 ms; during noun 0 ms to 471 ms; after_noun: > 471 ms). Overall, conditions differ considerably. While the *pair* condition (red, left) shows the strongest effects at occipital channels before mean noun offset (more negative for later mean target fixation time), the *singleton* condition shows more sustained effects from ~ 125 ms to ~ 625 ms with reversed polarity (more positive for later mean target fixation times), but similar topographic distribution (occipital ROI). In addition, the *singleton* condition shows a clear negative effect over central ROIs (more negative with later mean target fixation times) that is basically absent for the *pair* condition. The posterior and occipital positivity and (pre-)frontal negativity found for the *pair* condition at ~ 500 ms is also affected by the mean target fixation time: with later fixations, the positivity is stronger, but it does not allow a differentiation between fixation times *during* and *after* the critical nouns.

To summarize the results, we found that the pair condition elicited a distinct pattern of ERP effects, including a sustained frontal and posterior positivity peaking around 500 ms, a frontal negativity over right-hemispheric sites, and early occipital negativities, all largely absent or reduced in the singleton condition. Additionally, fixation timing modulated these effects differently across conditions: in the pair condition, later fixations amplified a posterior positivity, whereas in the singleton condition, later fixations led to a more sustained occipital positivity and central negativity.

**Fig. 6:**
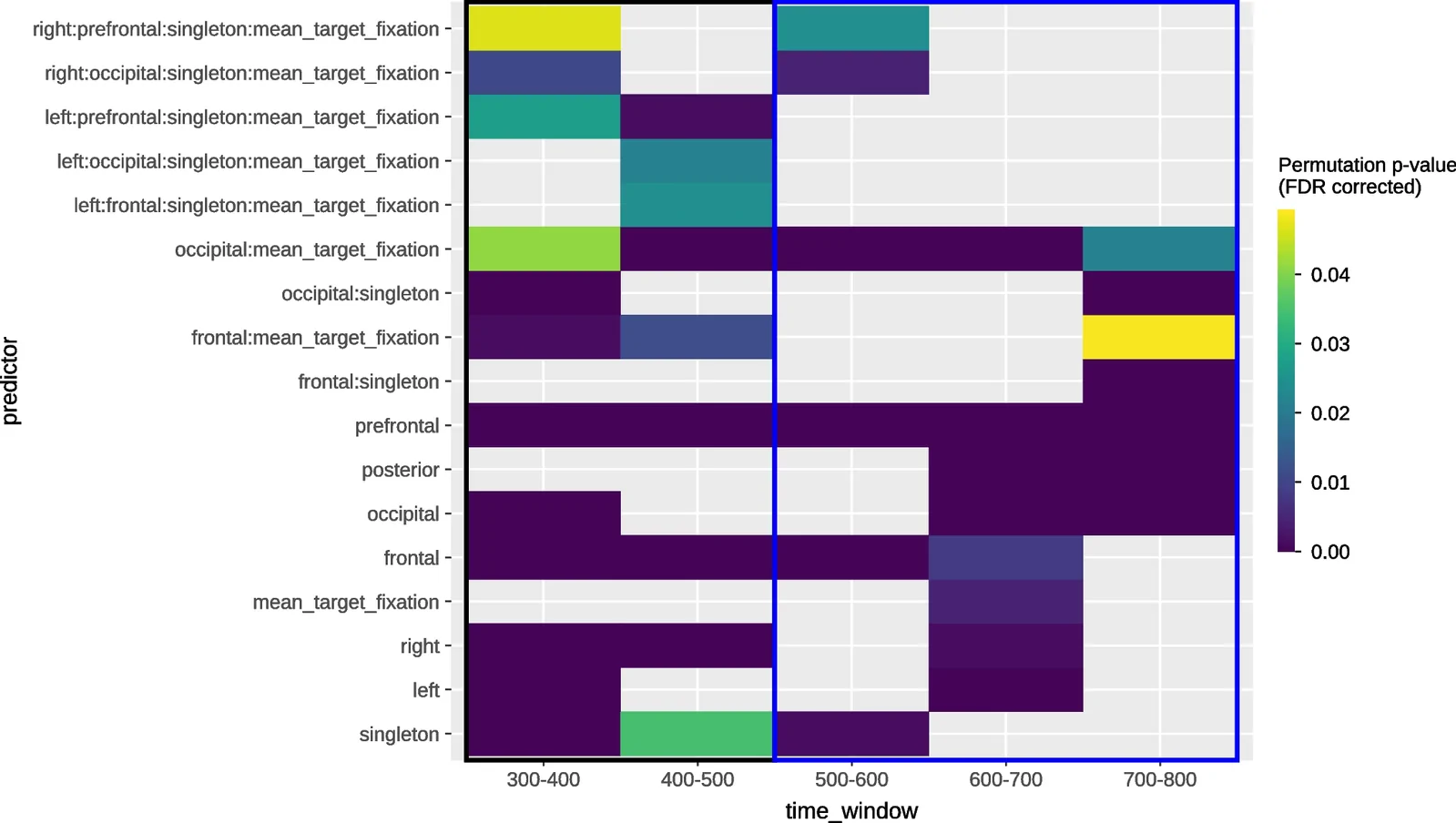
Significant terms as revealed by the permutation analysis for the language-related event-related potentials

Figure 6 shows a selection of significant effects in the time bins within the N400/P300 (black rectangle) and late positivity (blue rectangle) time range (see supplementary materials for a full graphical summary of the permutation results and the model summaries of the linear models per time bin). We see that the effect of *mean target fixation* significantly interacts with condition and with the topographical factors in both time windows, but with a focus on the earlier P300/N400 time window.

**Fig. 7:**
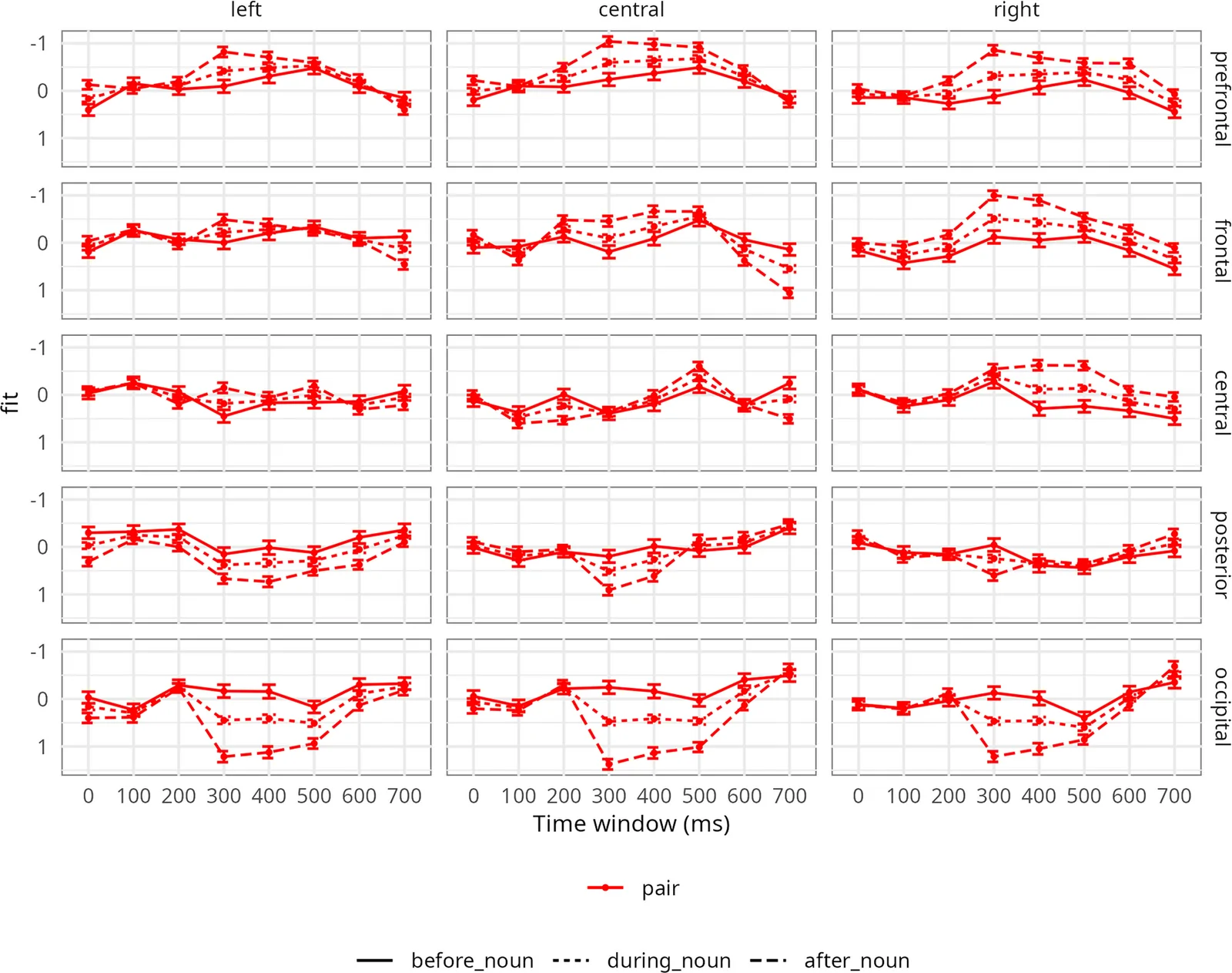
Effects of mean target fixation time in 100 ms time bins from 0–800 ms after noun onset for the pair condition. Error bars represent 83 % confidence intervals, corresponding roughly to 0.05 significance threshold. The continuous predictor was grouped for plotting purposes

**Fig. 8:**
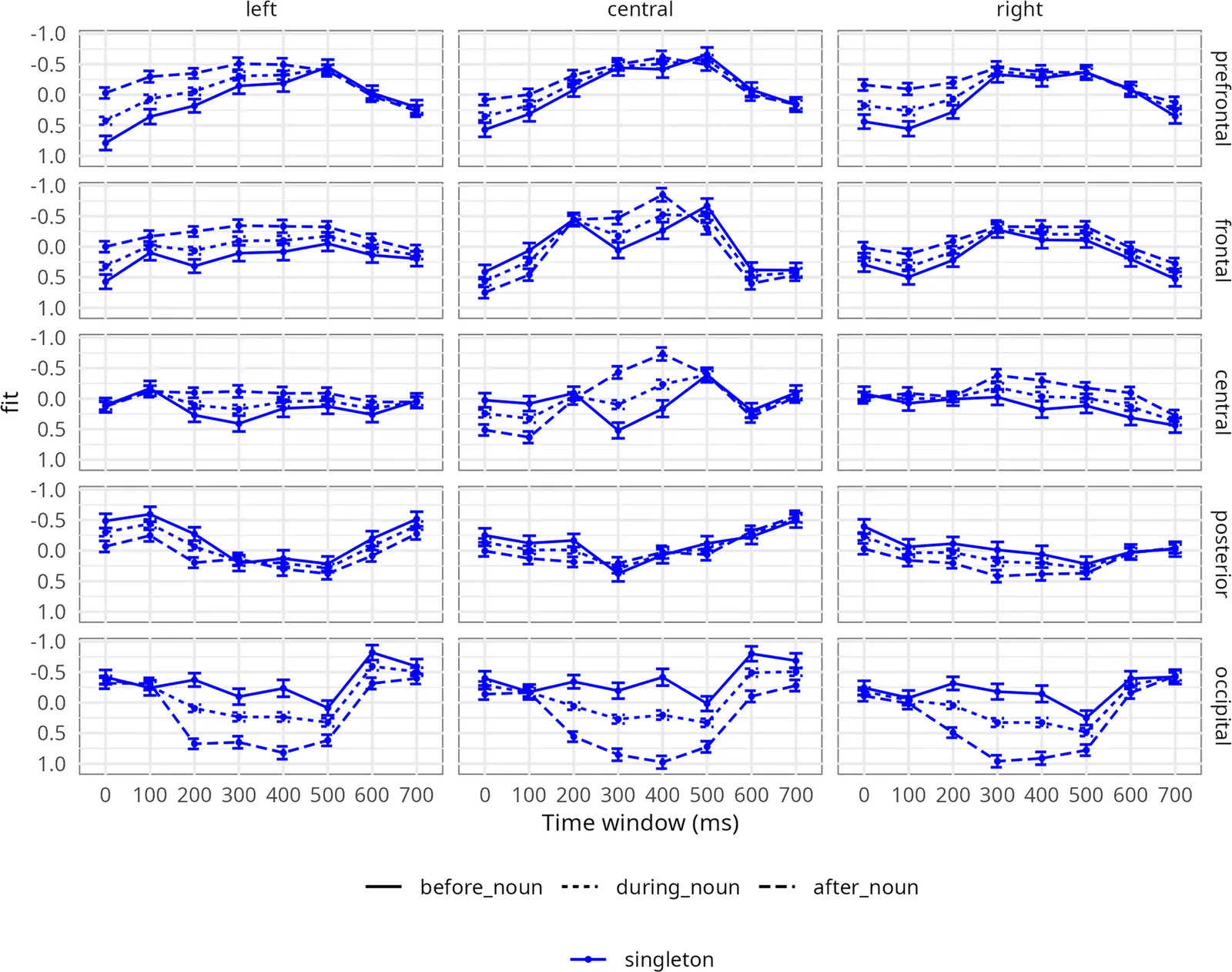
Effects of mean target fixation time in 100 ms time bins from 0–800 ms after noun onset for the singleton condition. Error bars represent 83 % confidence intervals, corresponding roughly to 0.05 significance threshold. The continuous predictor was grouped for plotting purposes

The effect plots of the time-range from 0 to 800 ms following noun onset (Figs. 7 and 8) make the time and topographic distribution of the significant effects a bit clearer. It becomes obvious from the effect plots in Fig. 7, that the effect of *mean target fixation* for the *pair* condition is strongest within the time-range of the N400/P300 (300–500 ms), visible as a positivity over central occipital channels. It is reversed over frontal and prefrontal channels and followed by a negativity strongest over right-central, frontal, and prefrontal channels. Although the earlier onset and more sustained nature of the negativity may suggest two different underlying effects, we cannot determine on the basis of the present data whether the negativity and positivity are different effects or share an underlying dipole. Therefore, we will not discuss them separately. Note that the very early effects directly after noun onset are very much attenuated as compared to the ERPs, because the *baseline* predictor canceled them out. For the *singleton* condition (Fig. 8), the picture is very similar, although the occipital positivity starts earlier (200–300 ms) and is less pronounced (hence the main effect of *condition* visible in the ERPs). The same is true for the centro-frontal negativity, which starts very early for the *singleton* condition (0–100 ms) and is rather left-lateralized and stronger over the central ROI, as compared to the *pair* condition. In a similar way as the positivity, it is less pronounced than for the *pair* condition, explaining the main effect of *condition* visible over these ROIs and time windows in the ERPs.

### Fixation-related ERPs

**Fig. 9:**
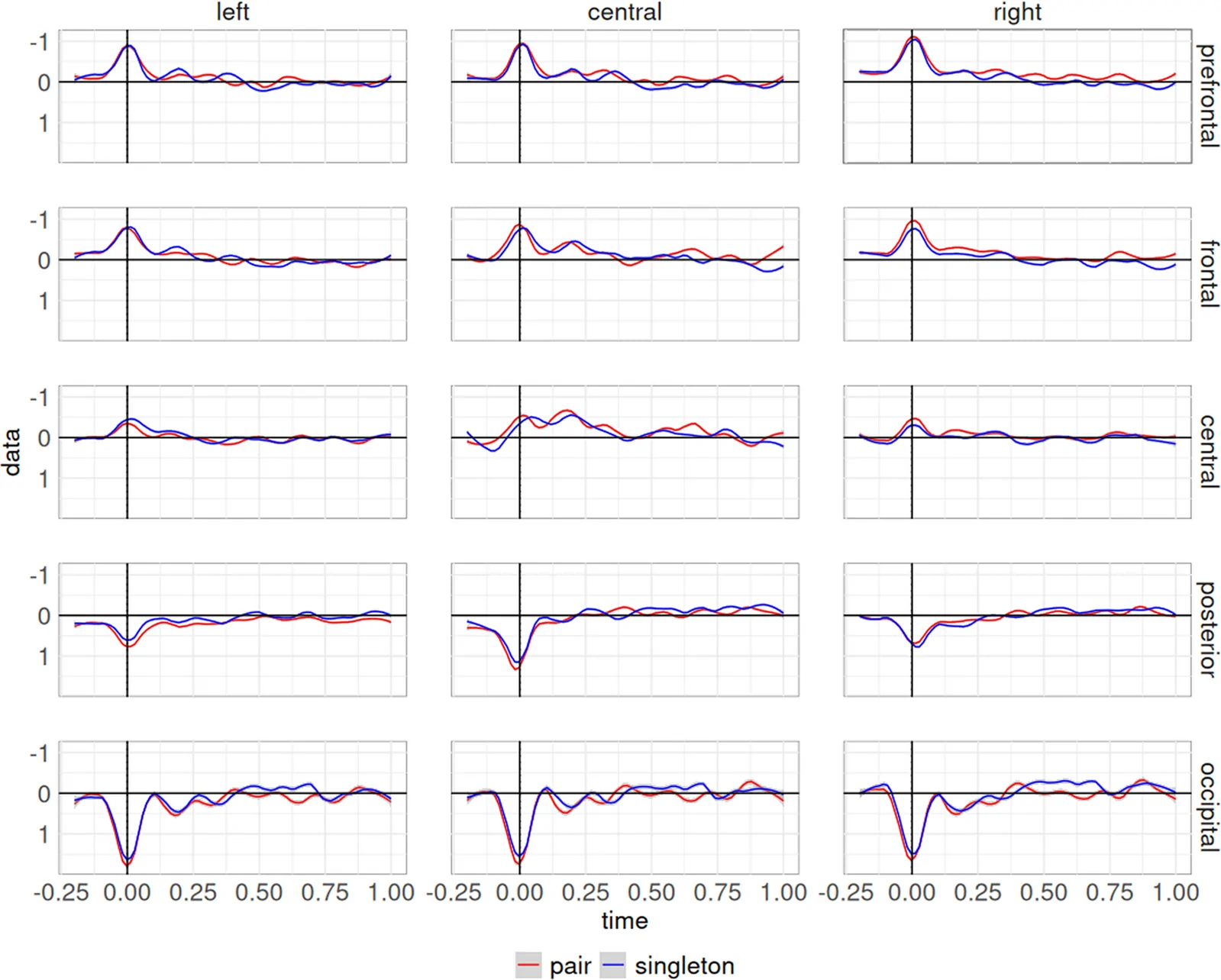
Fixation-related potentials from −200 to 1000 ms after fixation onset by condition and region-of-interest

**Fig. 10:**
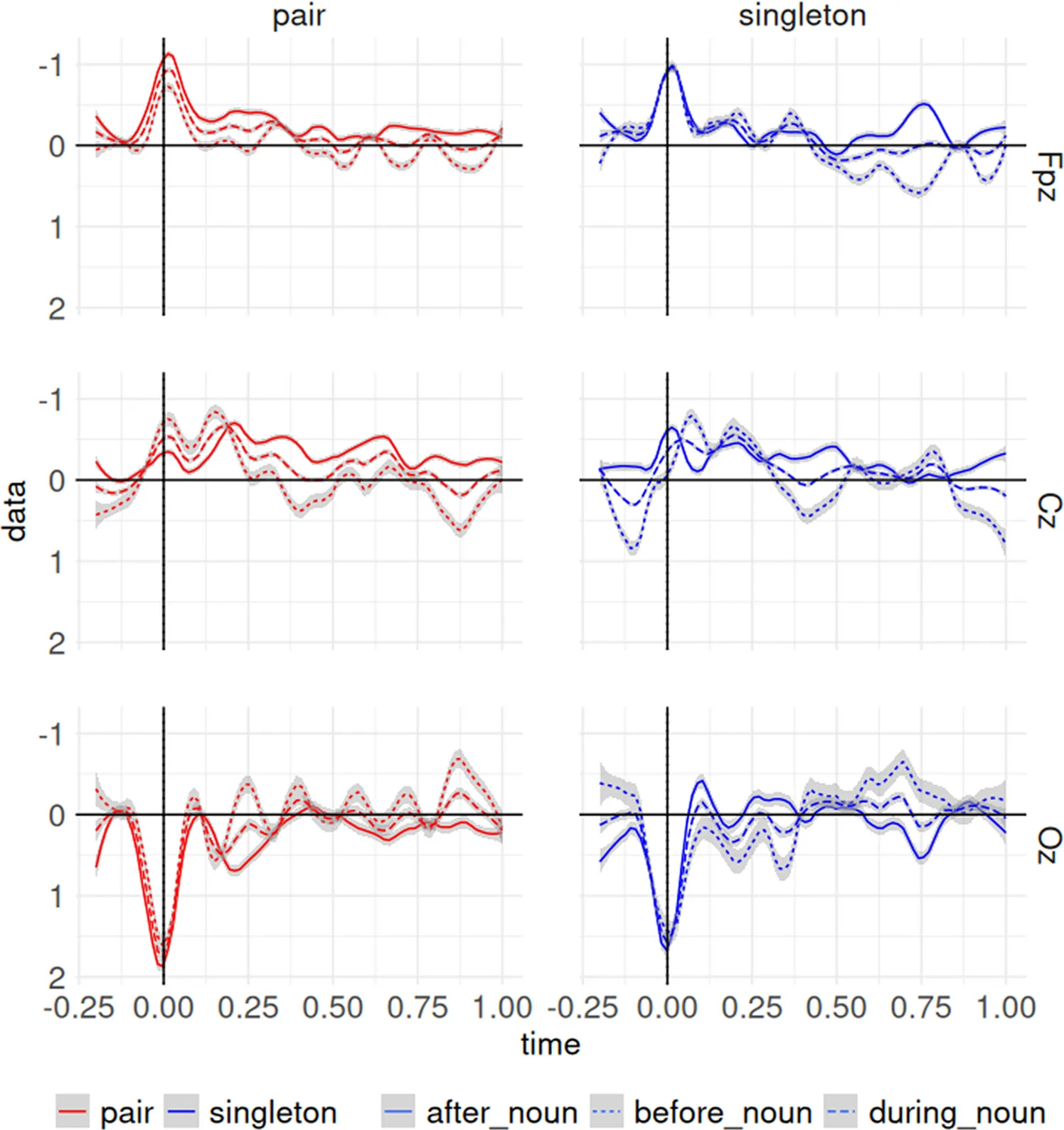
Fixation-related potentials of fixations to targets from −200 to 1000 ms after fixation by condition and fixation time at selected channels

Figure 9 shows the event-related potentials relative to *target* fixation onset by condition. The large positive deflection at 0 ms is probably the lambda response (Ries et al., 2018), which usually occurs 80–120 ms after fixation onset with stationary eye-tracking equipment. As of today, we have not identified the source of this latency shift, but we suspect it stems from the dispersion-based fixation detection algorithm, which assumes a dispersion of less than 1.2 degrees of visual angle for at least 80 ms, and may be the source of the latency shift. Combined with the limited accuracy of the mobile eye tracker (~ 1.5 degrees), this likely induced the shift. Future work could also account for head movements, in order to be able to move to a saccade-based detection algorithm as proposed by e.g., Engbert and Kliegl (2003). Except for a small positivity (~ 400–650 ms) for the *pair* conditions, there is almost no difference between conditions. However, if we turn to Fig. 10, which shows the fixation-related ERPs by fixation time at selected channels, we can see clear differences between the two conditions. First, the effect of fixation time starts very early for the *pair* condition at channel Fpz (0–250 ms), with a more pronounced positivity for earlier fixations (before > during > after noun). It is followed by and partly overlaps with two positive effects (~ 200–700 ms and ~ 740–1000 ms), which are strongest over Cz (with the same direction before > during > after noun). At channel Oz, there is a series of negative effects visible (before > during > after), which is less clear than the effects at channels Fpz and Cz. For the *singleton* condition, on the other hand, the effects are different. At channel Fpz there is only a rather late positivity for early fixations starting at ~ 500–1000 ms after fixation onset (before > during > after). It is preceded by a positivity over channel Cz between ~ 250 and 500 ms (before > during > after), which is similar to the effect found for the pair condition, but shorter (~ 250–500 ms). This positivity is followed by a later positive effect from ~ 800–1000 ms after fixation onset (after > during > before noun). At channel Oz, an early positivity is visible for the *singleton* condition (~ 100–375 ms), which is stronger for earlier fixations (before > during > after noun). It is followed by a later negativity (~ 500–800 ms) with a similar gradient (before > during > after noun).

Overall, the fixation-related potentials differed markedly by condition, with the pair condition showing earlier and more sustained effects of fixation timing across frontal and central sites, while the singleton condition exhibited later and more transient effects with distinct temporal and topographic profiles.

**Fig. 11:**
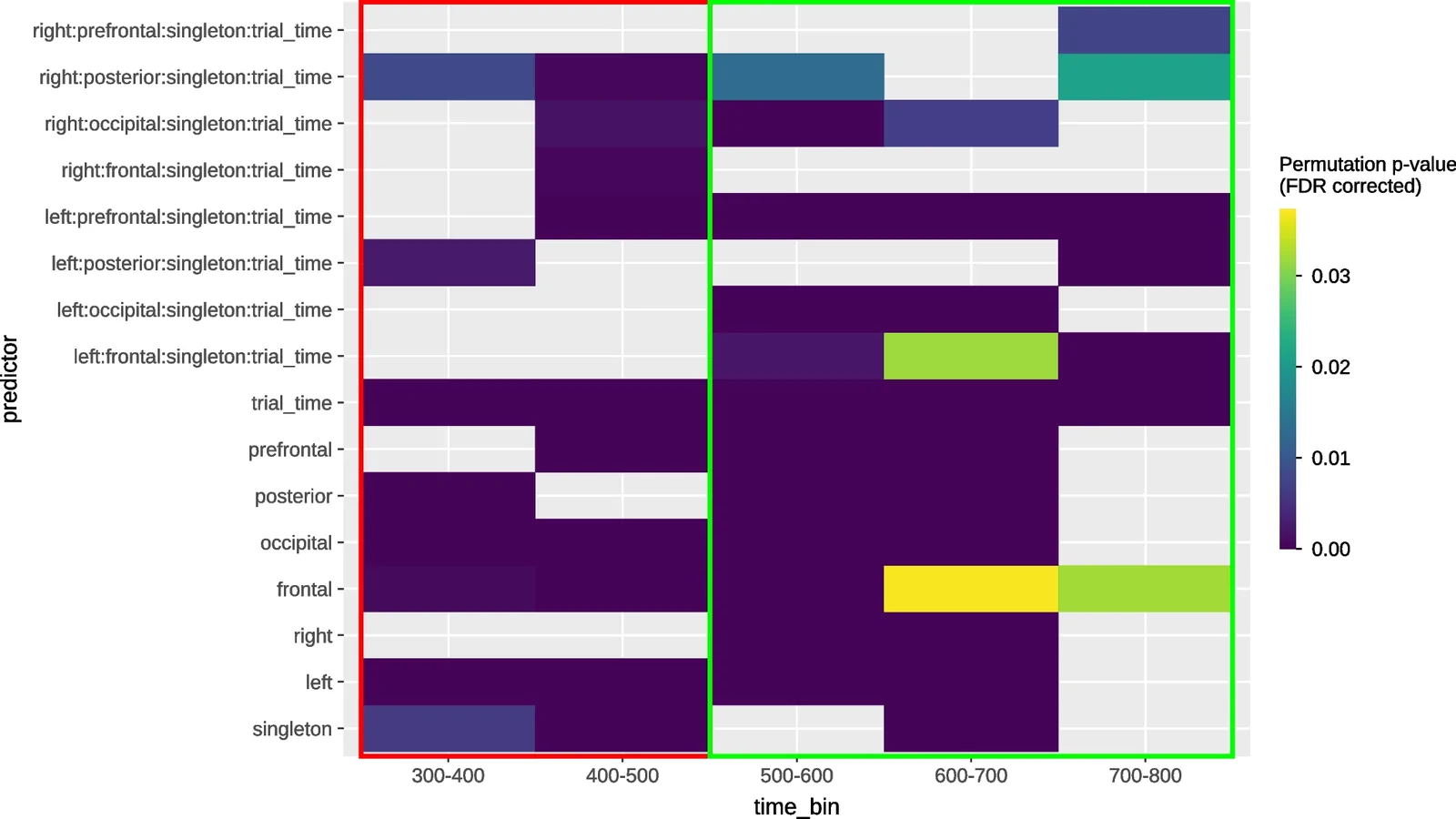
Significant terms as revealed by the permutation analysis for the fixation-related potentials

**Fig. 12:**
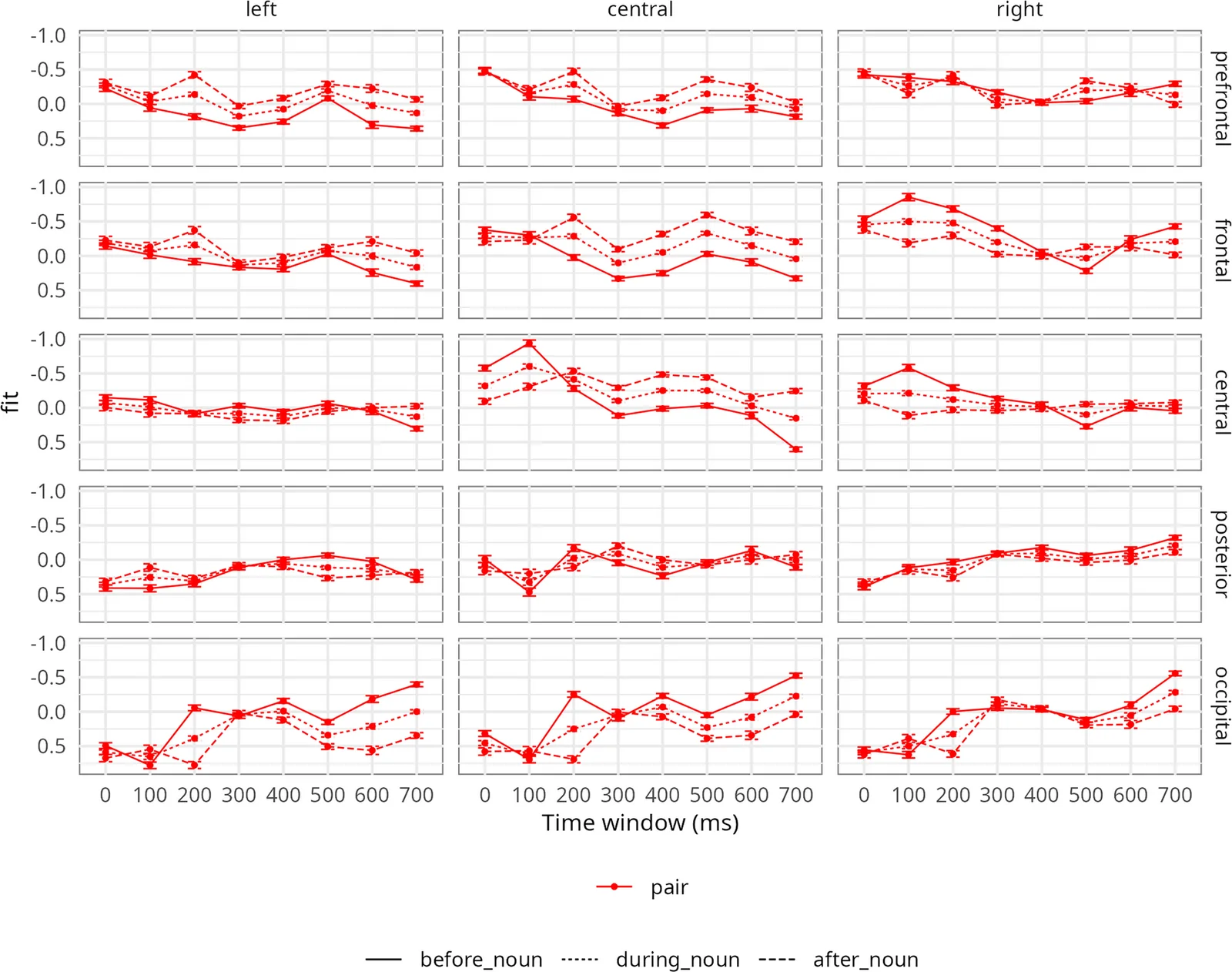
Effects of target fixation time in 100 ms time bins from 0–800 ms after fixation onset for the pair condition. Error bars represent 83 % confidence intervals, corresponding roughly to 0.05 significance threshold. The continuous predictor was grouped for plotting purposes

**Fig. 13:**
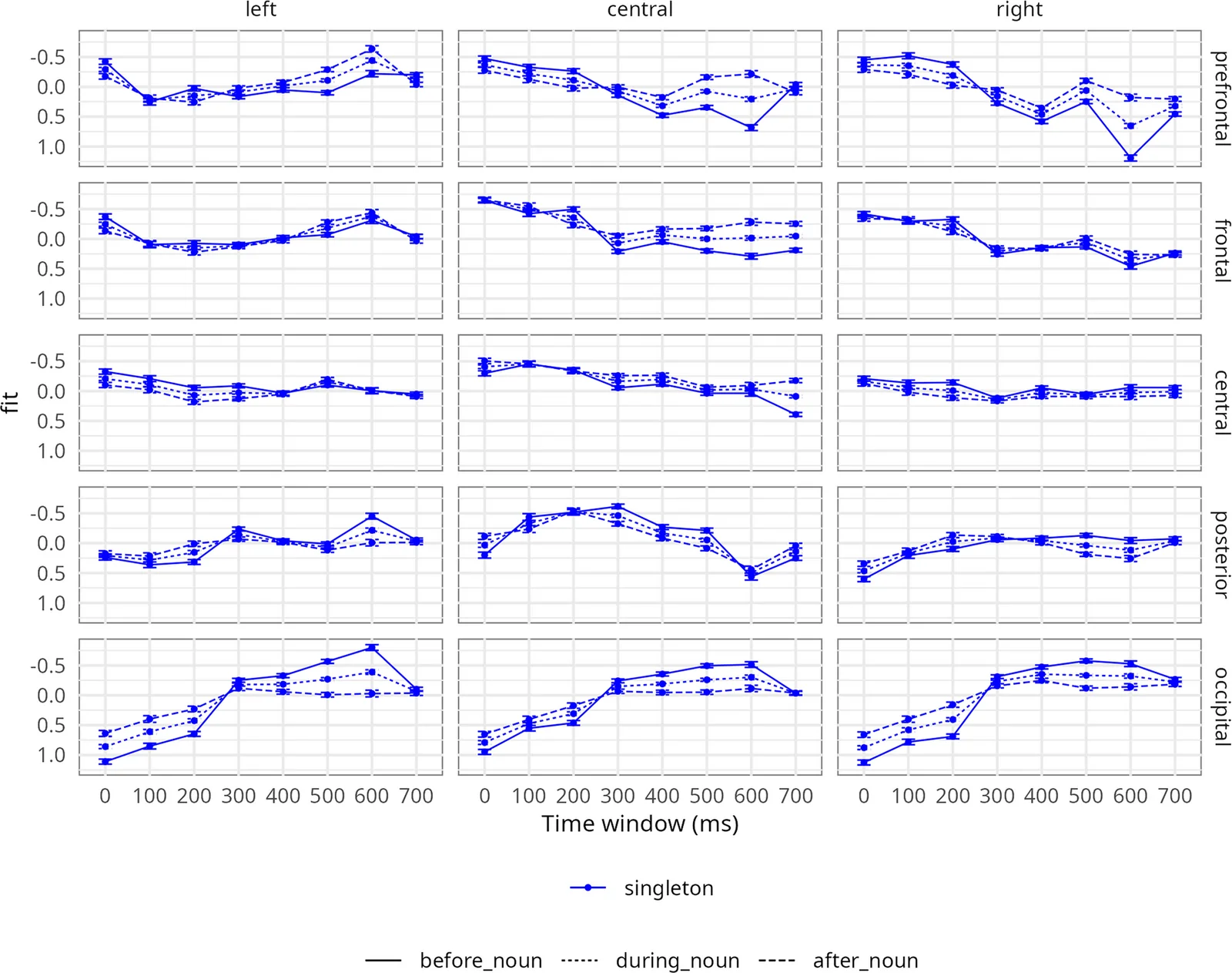
Effects of target fixation time in 100 ms time bins from 0–800 ms after fixation onset for the singleton condition. Error bars represent 83 % confidence intervals, corresponding roughly to 0.05 significance threshold. The continuous predictor was grouped for plotting purposes

Figure 11 summarizes the significant effects in the N400/P300 (300–500 ms) and late positivity time window (500–800 ms). Other than for the nouns, we can see that *trial time* significantly interacts with *condition* and the topographical factors in all time bins, without a clear focus on the earlier (N400/P300) or later (late positivity) time window. Figures 12 and 13 summarize the corresponding effects by ROI.

### Sensitivity analysis

**Fig. 14:**
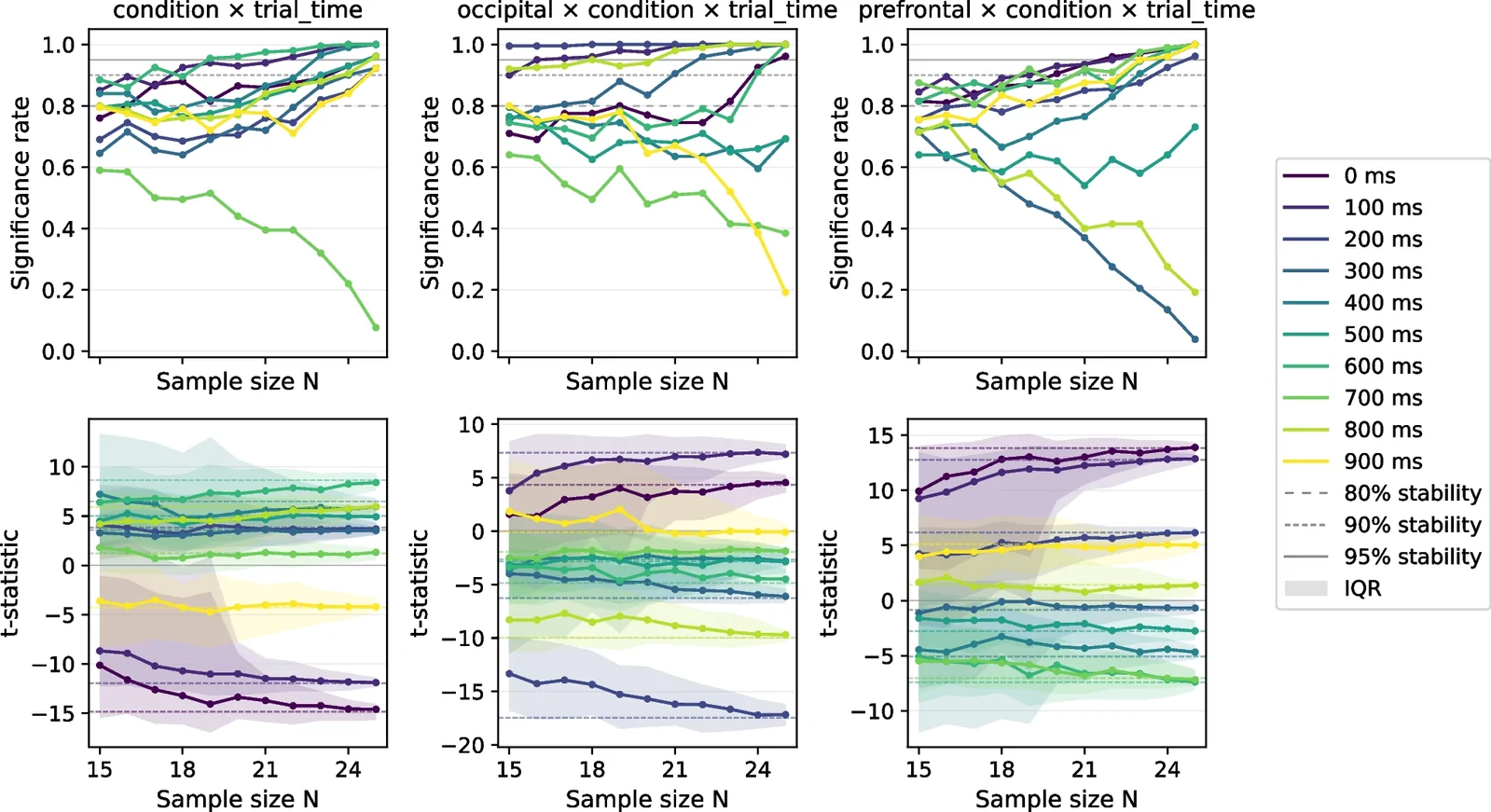
Results of the sensitivity analysis for fixation-related potentials. Upper panel: significance rate (alpha <= 0.05) for *N* = 15–25 at 80 %, 90 % and 95 % stability for selected model terms and time windows from 0–100 to 900–1000 ms. Lower panel: t-statistic stability for the effects by sample size and time window

**Fig. 15:**
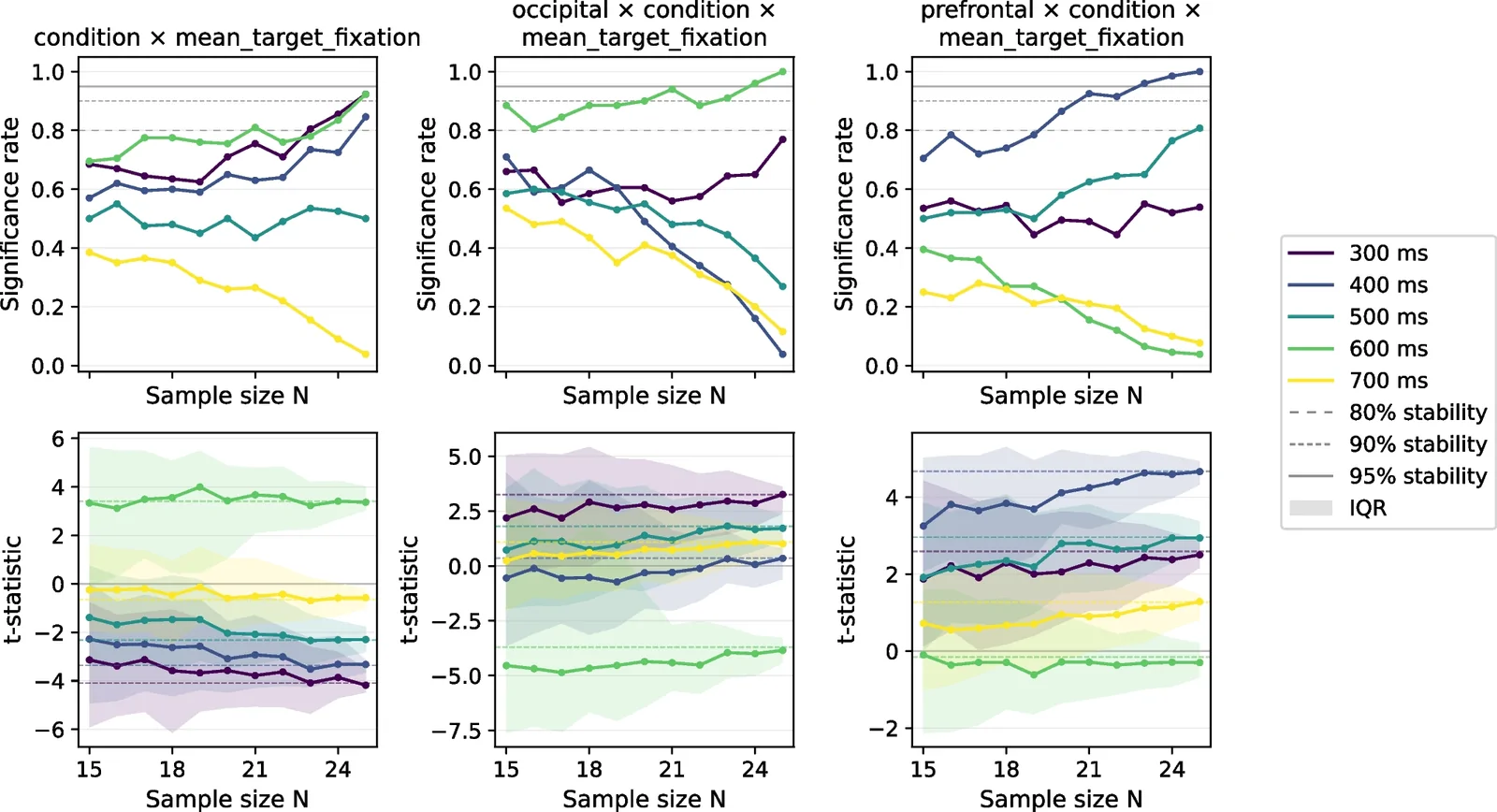
Results of the sensitivity analysis for language-related potentials. Upper panel: significance rate (alpha <= 0.05) for *N* = 15–25 at 80 %, 90 % and 95 % stability for selected model terms and time windows from 300–400 to 700–800 ms. Lower panel: t-statistic stability for the effects by sample size and time window

**Fig. 16:**
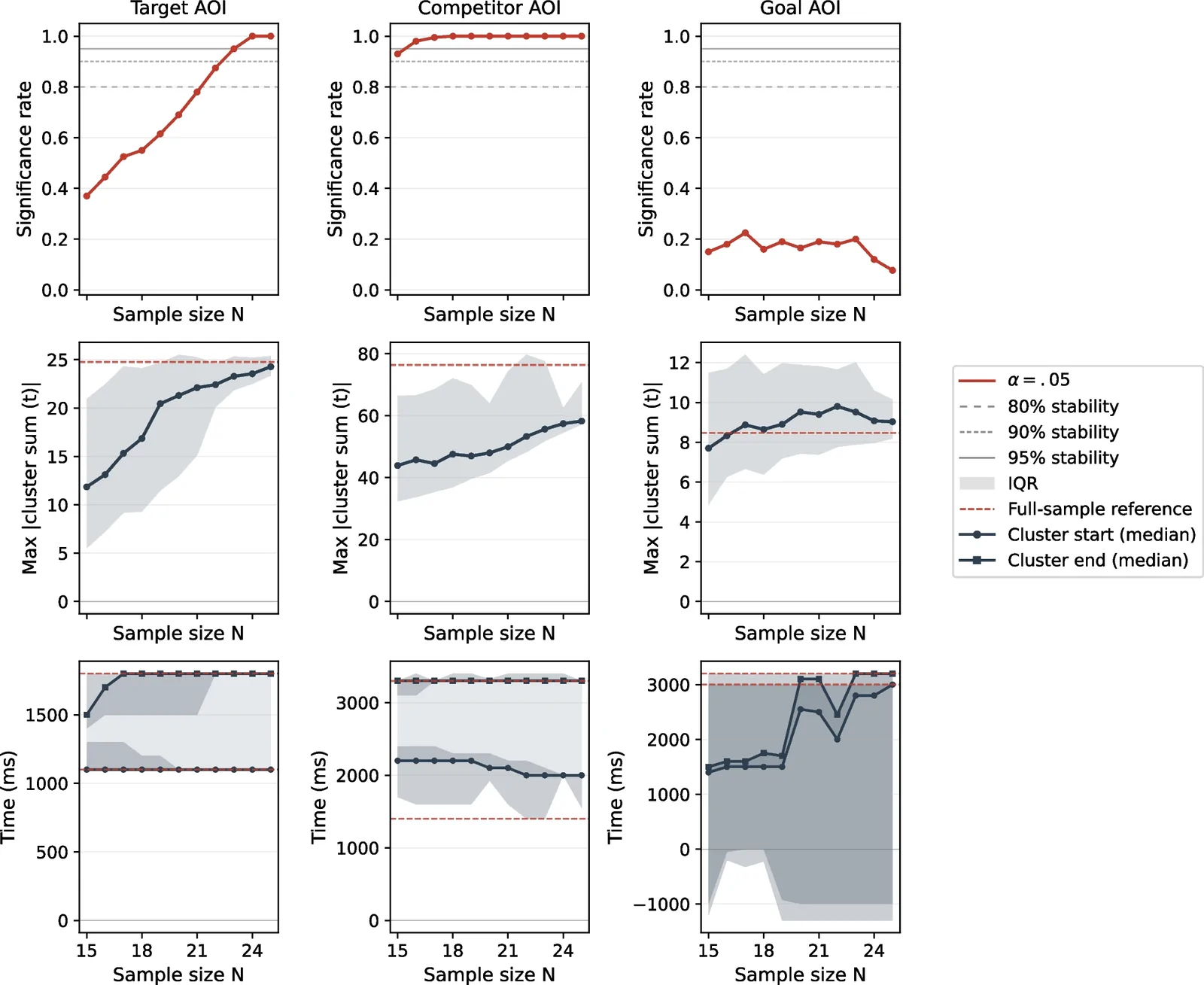
Results of the sensitivity analysis for the gaze proportions. Upper panel: significance rate (alpha <= 0.05) for *N* = 15–25 at 80 %, 90 % and 95 % stability for the target, competitor and goal AOI. Middle panel: maximum cluster sum for the AOIs. Lower panel: Cluster extent in milliseconds for *N* = 15–25

Figures 14–16 summarize the proportion of subsamples in which selected effects reached significance at α = 0.05, as a function of time (color) at different thresholds (80%, 90%, and 95%; see Supplementary Material at the OSF repository for full results). Each figure summarizes a subsampling analysis. For every sample size *N* (*x*-axis), we repeatedly drew random subsets of *N* participants from the full sample and re-estimated the model on each draw. The panels therefore do not show a single result but the distribution of results to be expected at that *N*. Two complementary quantities are plotted. The significance rate (top row) is the proportion of draws in which the effect reached significance at α = 0.05; it can be read as an empirical detection probability – the reliability with which the effect would be recovered at a given sample size. The horizontal lines mark the 80%, 90%, and 95% levels used as stability thresholds. The remaining row(s) show the stability of the estimate itself: the median statistic across draws, with the shaded band giving the interquartile range (IQR) over draws.

Read together, the two views distinguish a robust from a fragile effect. A robust effect reaches a high significance rate already at small *N* and then plateaus, while its estimate settles onto a stable median with a narrowing IQR. A fragile or underpowered effect climbs only slowly (or not at all), stays below the stability thresholds, or retains a wide IQR even at the largest available *N*. Because each colored line corresponds to one post-onset time bin (Figs. 14 and 15) or each subfigure to one area of interest (Fig. 16), the figures also reveal which time windows or regions carry the stable effect and which remain underpowered.

For the fixation-related potentials (Fig. 14), the condition * trial_time interaction – the central finding of our study – reached the 80% stability threshold in 9 of 10 time bins (Fig. 14, upper left panel), with a median threshold of *N* = 16; only the time bin centered at 700 ms did not reach the threshold at any tested *N*. The condition main effect on fixation-related potentials reached the same threshold in 7 of 10 time bins, at a median *N* of 23. Topographic interactions were quite stable at occipital ROIs (Fig. 14, middle panel) and at prefrontal ROIs (Fig. 14, upper right panel). At occipital sites, 6 of the 10 time bins crossed the 80% significance-rate threshold, their significance rates increased monotonically with sample size, and the corresponding *t*-statistic IQRs were narrow and settled from *N* ≈ 22 onward. For the prefrontal effects, 7 of 10 bins reached the threshold, and the *t*-statistic IQRs became narrower across the tested range. However, several time bins (300–400, 500–600, 800–900) showed significance rates that decreased with increasing *N* (e.g., the bin centered at 300 ms, whose detection rate fell toward zero at the largest *N*), the signature expected from an underpowered or unstable effect.

For the language-related potentials, the picture is more mixed. The condition main effect within the N400/P300 latency range (300–500 ms) reached the 80% stability threshold only in the 300-ms bin (threshold *N* = 21); the 400 and 500 ms bins did not stabilize at any tested N. However, the interaction condition * mean_target_fixation, which is central for our present proof-of-concept, stabilized in the 300, 400, and 600 ms bins (Fig. 15, threshold *N* = 21–25), but not in the 500 ms or 700 ms bins. We read this as evidence that the language-related effects in our data lie closer to the lower detectability boundary at the present sample size than the fixation-related effects, and we treat them with corresponding caution in the discussion below. Topographic patterns were broadly consistent with the second-order analysis, with posterior and occipital effects stabilizing at smaller *N* than prefrontal effects.

For the gaze data (Fig. 16), the picture is reassuring. The competitor effect (fewer looks to privileged ground competitors in the *pair* than to other non-target objects) was extremely robust: the cluster reached significance in 93% of subsamples already at *N* = 15 and in 100% of subsamples at every *N* ≥ 18; threshold N at the 80% stability level is 15. The target effect (more looks to targets in the *pair* than in the *singleton* condition) showed a graded pattern characteristic of a moderate effect size, with the significance rate climbing monotonically from 37% at *N* = 15 to 78% at *N* = 21, 88% at *N* = 22, and reaching 100% at *N* = 24; threshold N at the 80% stability level is 22, and at the 95% level it is 23. The goal effect, which was not significant in the full sample (*p* = 0.13), did not reach the 80% stability threshold at any tested *N* at α = 0.05 or stricter, in line with the absence of a population-level effect.

Taken together, the sensitivity analysis indicates that the gaze and fixation-related effects we report are robust to substantial reductions in sample size: the competitor effect on gaze proportions is detectable across the entire tested range, the target effect on gaze proportions stabilizes at *N* = 22, and the key condition * trial_time interaction in the fixation-related potentials stabilizes well below our final *N* of 26. The language-related potentials yield a clear primary result: the N400 effect in the 400–500-ms window stabilized robustly, crossing the 80% threshold at moderate N and remaining stable across the tested range. The pattern across the remaining bins is more uneven – the earlier part (300–400 ms) of the N400/P300 window, together with the higher-order interactions across both ERP types, did not stabilize consistently within the tested range, especially for the higher-order interactions. The stability of the condition * mean_target_fixation N400 effect (400–500 ms) indicates that our central finding lies well within that resolvable range.

### Summary

#### Gaze data

The analysis of the gaze proportion revealed a significant increase in looks to the target object in the presence of a referential competitor in privileged ground (*pair* condition). Competitors, on the other hand, received significantly fewer looks than all the other non-target objects in the visual display. The numeric increase in looks to the goal compartment for the *pair* condition did not reach significance. We assume that the absence of looks to the competitor objects can be explained via attentional mechanisms: As we discussed in the introduction, the target objects are the most informative objects with regard to solving the experimental task. First of all, they fit the meaning of the linguistic utterance (e.g., being a vase), second, they provide all information necessary for resolving the referential ambiguity (hidden or not), and, most importantly, they are the target object that is intended to be moved. Because the hidden competitors remained in place for an entire experimental block (10–15 minutes), while the target objects changed position every few trials, participants likely learned where the target objects were not located and paid little attention to the hidden objects. However, why the target objects in the *pair* condition received more looks than the target objects in the *singleton* condition is hard to explain without additional data. Fortunately, fixation-related potentials can clarify this.

#### Fixation-related potentials

In the present study, the amplitude of the fixation-related potentials time-locked to target fixations was not directly influenced by condition in the time windows of interest (300–500, 500–800 ms). Both targets in the *singleton* and *pair* conditions elicited very similar ERPs with only minor differences. Still, we have reason to assume that participants treated the “*pair* targets” differently, as compared to the “*singleton* targets”, since differences in the fixation related potentials become apparent, when we consider the time in a trial at which a fixation occurred: when more linguistic evidence was available that the object currently looked at is the actual target object (fixations during or after the noun), fixations to targets in the *pair* condition elicited an early occipital positivity (200–300 ms), followed by a sustained positivity with a similar gradient response. This early positivity was absent in singleton trials, which only showed a sustained positivity at occipital channels. Both effects, the early positivity for *pair* trials and the sustained positivity for both conditions, are mirrored over central, frontal, and prefrontal electrodes. We assume that the amount of available linguistic information is responsible for the graded effects of fixation time. Fixations that occurred during (partial information available) or after the noun (all information available) elicited more positive deflections in the fixation-related potentials over posterior and occipital channels, and vice versa over frontal and prefrontal channels, because the visual information provided by the target object matches the linguistic information provided by the word. The fact that the effect is stronger in the *pair,* along with the *pair* specific early positivity, strengthens our assumption that targets in the *pair* attract attention and are, in a certain sense, treated as “targets among targets”.

#### Language-related potentials

The language-related potentials time-locked to noun onset revealed a positivity for the *pair* condition in the posterior and occipital ROIs. In central, frontal, and prefrontal ROIs the amplitude of this effect reversed, while its temporal and spatial distribution remained rather constant. The statistical analysis returned significant condition effects within both the N400/P300 (300–500 ms) and the later time window (500–800 ms) on which we focused our analysis. As reported in the sensitivity analysis above, the stability of these effects is uneven across time bins: the N400 effect in the 400–500 ms window stabilizes robustly, whereas the earlier part of the window (300–400 ms) and the higher-order interactions do not stabilize within our tested range. We therefore read the language-related pattern as suggestive rather than firmly established at the present sample size, and return to this point in the discussion. We assume that this positive deflection is a P300 related to attentional reorientation following the detection of a linguistic target. It is reduced for targets in the *singleton* condition, since they do not have referential competitors and are thus easier to identify by virtue of our experimental design. Moreover, target words for which the majority of fixations to the target object occurred before or during hearing a target noun, i.e., trials in which participants either seemed to have made rather accurate predictions of the upcoming object (most looks before noun) or have collected enough (partial) linguistic information to make a prediction (most looks during noun), show a decreased positivity in both conditions, although this effect is less strong and starts earlier in the singleton condition. Overall, this likely shows that the P300 reflects the degree of attentional reorientation necessary to solve the experimental task: identifying the target object. When the target object’s location is already known (before noun), the P300 is maximally reduced, whereas in trials in which participants still have to actively search for the object after or during hearing the noun, target detection is more difficult and requires more attentional resources, because participants have to initiate a visual search, hence shift from a perceptive to an action-based attentional state.

## General discussion

We presented results from an interactive, real-world study that used mobile EEG and eye-tracking equipment to simultaneously record eye-tracking and EEG data from participants engaged in the director task. With as few instructions for the directors as possible, we tried to create an experimental situation that is as natural as possible given the experimental task and its repetitive nature. We discussed and demonstrated how difficulties with event-related potentials in real-world EEG studies can be overcome using analysis tools suited to this kind of data, and how multimodal data recordings (EEG, ET) can be reciprocally informative. We showed that the temporal distribution and availability of visual (gaze) and speech information affect both fixation-related *and* language-related event-related potentials. Together with the behavioral gaze data, multimodal data recording provides a relatively complete picture of participants’ visual and auditory attention, allowing us to draw conclusions about one modality that are informed by the other. Because visual and auditory information are virtually always present simultaneously in real-world interaction, our results highlight the importance of analyzing them together when working with data from real-world, interactive experiments. Next, we discuss our results against the literature and offer suggestions for further exploring real-world communicative interaction.

### Gaze distributions as cognitive offloading

In our experiment, participants showed an increase in the proportion of looks to the target object in trials with a referential competitor in privileged ground (*pair* condition). This outcome is at odds with our initial prediction that competitors would be attended to as informative cues for perspective-taking. Instead, the data suggest that in our simplified design, participants adapted their strategy by prioritizing direct information from targets and disregarding competitors, which provided no additional task-relevant information once their occlusion status was known. The results also differ from the previous ERP studies (Sikos et al., 2019; Richter et al., 2020), which both found a reduction in target looks for referents with a privileged ground competitor. Still, they are in line with other studies demonstrating that the strength of the interference effect of referential competitors depends on a variety of factors, including (linguistic) context (e.g., Heller et al., 2008; Brown-Schmidt et al., 2008), task (Ferguson et al., 2015; Ryskin et al., 2020), culture (Wu & Keysar, 2007; Wu et al., 2013; Wang et al., 2019), and the availability of cognitive resources (Epley et al., 2004a; Cane et al., 2017). Clearly, our very simple experimental design and the fact that competitors remained in their compartment for the entire 10–15 min of a block made the referential conflict very explicit. Participants likely learned certain aspects of the placement of objects, so that “ignoring” the referential competitors was possible without increasing the effort of solving the task. Moreover, our use of *identical* competitors also likely reduced the number of looks to competitor objects further, since there was no information gain from looking at the competitors, as compared to looking at the target objects. As we discussed above at several points, the target objects in the *pair* condition were the most salient and most informative stimuli in terms of task resolution, since they provide all necessary information for task resolution *and* they are the target object that is supposed to be moved. This makes looking at the competitor object (whose location participants were aware of) uneconomical. This is different from Richter et al. (2020) or Sikos et al. (2019), where participants had to compare sizes or accessories of referential candidates in ever-changing, randomized visual displays to reach a decision. We argue that this is a behavior called cognitive offloading (Risko & Gilbert, 2016): participants look at the stimulus display to gather the information that is necessary for task performance, since the alternative, memorizing the array, is cognitively very demanding. While this likely happened in Richter et al. (2020) and Sikos et al. (2019), for the reasons outlined above, such a behavior was not necessary and even infelicitous in the present study, since learning certain aspects of the visual display was connected with less effort in the present study.

Further evidence for our claim that targets in the *pair* condition require more attentional resources in terms of reorientation comes from the effects in the fixation-related potentials. Previous research on fixation-related potentials has identified a P300 effect for fixation-related potentials, that distinguishes between fixations on singleton target stimuli within a display of distractors and fixations on the distractors (Brouwer et al., 2013; Kamienkowski et al., 2012). This is also supported by strong evidence from intracranial recordings, showing that the brain dissociates between target and distractor stimuli at the single-neuron level (Wang et al., 2018). The present fixation-related potentials show very early positive responses following target fixations in the *pair* condition, given that (partial) linguistic information has already been provided. That is, fixations that occurred during or after the target noun has been uttered elicit a stronger early positivity effect in the *pair* condition, but not in the *singleton* condition. This provides indirect evidence that, compared to targets in the *singleton* condition, participants seemed to attribute greater attentional resources to the targets in the *pair* condition. This finding in the fixation-related potentials helps us in the interpretation of the gaze behavior and the language-related event-related potentials, as we can make assumptions about participants’ attentional states that are informed by data acquired from the participants themselves.

### The N400-P300 complex and multimodal integration

In the language-related potentials, we found a P300 following nouns, with higher amplitude in the *pair* condition. It is thus in line with the ERPs in Richter et al. (2020) and Sikos et al. (2019), who also found increased positivity for referents with common-ground competitors. However, given that we found the reverse effect in the gaze proportions for targets, as compared to their study, this is surprising. The finding therefore suggests that P300 amplitude is not straightforwardly tied to the *number* of target fixations, but rather reflects the detection of referential conflicts that require attentional reorientation. Given the nature of the P300 as a decision related brain potential that seems to be at the intersection of cognition and behavior (e.g., Verleger et al., 2005; Nieuwenhuis et al., 2005, Aston-Jones & Cohen, 2005; Aston-Jones & Waterhouse, 2016; Chennu et al., 2009; Jung et al., 2001; Makeig et al., 2004; Marathe et al., 2013, O’Connell et al., 2012; Townsend et al., 2001), the reduction in amplitude for the *singleton* targets becomes readily explainable: the target object can be understood similar to the repeated presentation of a stimulus in a controlled experiment (e.g., a standard): the *singleton* targets can be understood as the unsurprising, predicted and easily identifiable “standard” and the *pair* as the surprising, harder-to-detect deviant, requiring attentional reorientation and more effort (i.e., more looks) to be identified and selected correctly. As a consequence, they elicit the stronger P300 response. This interpretation is further strengthened by the effect of mean target fixation time for both conditions: the later participants fixated on the target, i.e., the longer they needed to identify it, the larger the amplitude of the P300 in both conditions. The fact that, e.g., Richter et al. (2020) report fewer looks to the target in their conflict condition is, from our perspective, explainable by cognitive offloading (Risko and Gilbert, 2016): in Richter et al. (2020), participants compare sizes of distractors and targets in order to arrive at a decision. By contrast, in our design, participants solve the more difficult task by looking more frequently at the actual target, since the distractor is uninformative and its position is known. In both cases, participants use their gaze to collect the evidence needed to reach a decision, i.e., they employ the same action with different strategies. In other words, the differences that appear to exist between our studies are not real differences.

Overall, the same N400/P300 time window sensitive to multimodal integration in controlled laboratory studies is also informative in our interactive, real-world setting. The weight of this argument rests on the data streams that our sensitivity analysis identifies as robust: the gaze data, the fixation-related potentials, and – for the language-related potentials – the N400 effect in the 400–500 ms window, all of which stabilize well below our final sample size. In each case, responses in the N400/P300 range track the time at which participants fixated the target object, mirroring natural reading, where semantically congruent parafoveal preview gradually reduces the P300/N400 amplitude of the following fixation (Antúnez et al., 2022; Dimigen et al., 2012; Li et al., 2024; Niefind & Dimigen, 2016). To this extent our results converge with previous work reporting multimodal-integration effects in the N400 time window (e.g., Özyürek et al., 2007; Peeters et al., 2015; Willems et al., 2008; Zhang et al., 2021) and with Kutas and Federmeier's (2011: 22) characterization of the N400 as “[a] region [that] is more accurately described as reflecting the activity in a multimodal long-term memory system that is induced by a given input stimulus during a delimited time window”, highlighting the need for a multimodal view on language and the brain, as put forward by Hasson et al. (2018).

We want to be explicit, however, about how far the language-related potentials can carry this interpretation at the present sample size. The same sensitivity analysis that confirms the 400–500 ms N400 shows that the earlier part of the window (300–400 ms) and the higher-order interactions do not stabilize well within the tested range. We therefore treat the broader reading – that interlocutors recruit multimodal information to reach referential decisions, and that this integration is reflected across the N400-P300 complex – as a hypothesis made plausible by our robust gaze and fixation-related findings rather than as a claim the noun-locked ERPs establish on their own. The finer-grained language-related pattern points in the same direction, but remains to be confirmed at the sample sizes our sensitivity analysis indicates.

## Conclusion

With the current study, we demonstrated that the real-time dynamics of language in interaction can be investigated using mobile recording equipment. Even under naturalistic conditions, with participants moving their upper body and large amounts of experimental noise, it is possible to calculate event-related potentials from noisy EEG data, with effects of multimodal integration showing in the N400/P300 time window, in line with what has been repeatedly demonstrated in controlled studies. Overall, our results required us to revise several of our original assumptions. For instance, although we predicted that competitors would be attended as informative cues, participants in fact ignored them; although we predicted that pair targets would be easier, the data revealed stronger P300 effects indicating greater attentional demand. These deviations from our predictions are not contradictions but highlight how real-world data can challenge and refine theoretical expectations. We want to stress at this point that it is not necessary for this purpose and, given the multimodal nature of our stimuli, not really adequate, to offer isolated linguistic explanations for our results. As pointed out by Hasson et al. (2018), we showed that it is possible to explain the present results with reference to domain-general processes related to attention and memory (learning of distractor positions in shelf, cognitive offloading, attention) and prediction (pre-noun looks to target objects, semantic prediction), alone. At least for this kind of linguistic input (very simple, reduced utterances with noun phrases and without verbs), it is not necessary to draw back to language-specific brain functions that should or should not be reflected in the N400 time window.

A potential limitation concerns object placement between blocks: rather than following a pre-generated randomization list, trained lab assistants rearranged the shelf ad hoc within the design constraints. While the assistants were blind to the study hypotheses and had no access to the recorded data, future studies adopting this paradigm should use pre-generated, documented randomization schemes to rule out unintended regularities in object placement fully.

Given the scarce amount of eye tracking and EEG data on unconstrained communication, we evaluated the validity of our results using sensitivity analysis, which has practical implications for researchers wishing to adopt this workflow. The robustness of the central fixation-related effect at sample sizes well below our final number of analyzed datasets suggests that comparable proof-of-concept studies are feasible in the range of *N* = 20–25 fully recorded participants, given the same number of trials per participant and a similarly homogeneous design – at least for fixation-related effects of comparable magnitude. For language-related effects of the kind we report here, our sensitivity analysis suggests that this range is closer to the lower limit of what is required, and we would recommend planning conservatively above it. However, we want to caution that this estimate is specific to our paradigm, our exclusion rate (which was largely equipment-driven), and the relatively constrained linguistic structure of the director task. For paradigms with greater linguistic variability, fewer trials per participant, or weaker condition contrasts, the trade-off between per-participant data quality (more trials, longer recording) and number of participants will shift. A simulation-based power analysis framework would make this trade-off explicit and would allow prospective sample-size planning for future studies in this analytic framework.

More broadly, our work fits into a growing body of multimodal EEG studies that move beyond the traditional screen-based paradigm. Recent work using immersive virtual reality has, for instance, shown that even subtle differences in socio-communicative intent (declarative, informative, imperative) translate into distinguishable electrophysiological signatures already at early planning stages of pointing gestures (Raghavan, Raviv, & Peeters, 2023). The present study takes a complementary, real-world (rather than virtual) approach: instead of immersing participants in a controlled 3D environment, we record from two interlocutors in a face-to-face dyadic setting. Both approaches aim to capture the multimodal richness of human communication while preserving EEG temporal resolution.

Rather than offering a comprehensive theoretical account, this study should be understood as a methodological proof of concept. Its primary contribution is to demonstrate that deconvolution-based regression ERPs can be reliably estimated from naturalistic interactive EEG data, that fixation- and language-related potentials can be informatively related, and that the full analytic workflow is reproducible. We believe these contributions stand on their own at the present sample size.

**Fig. 17:**
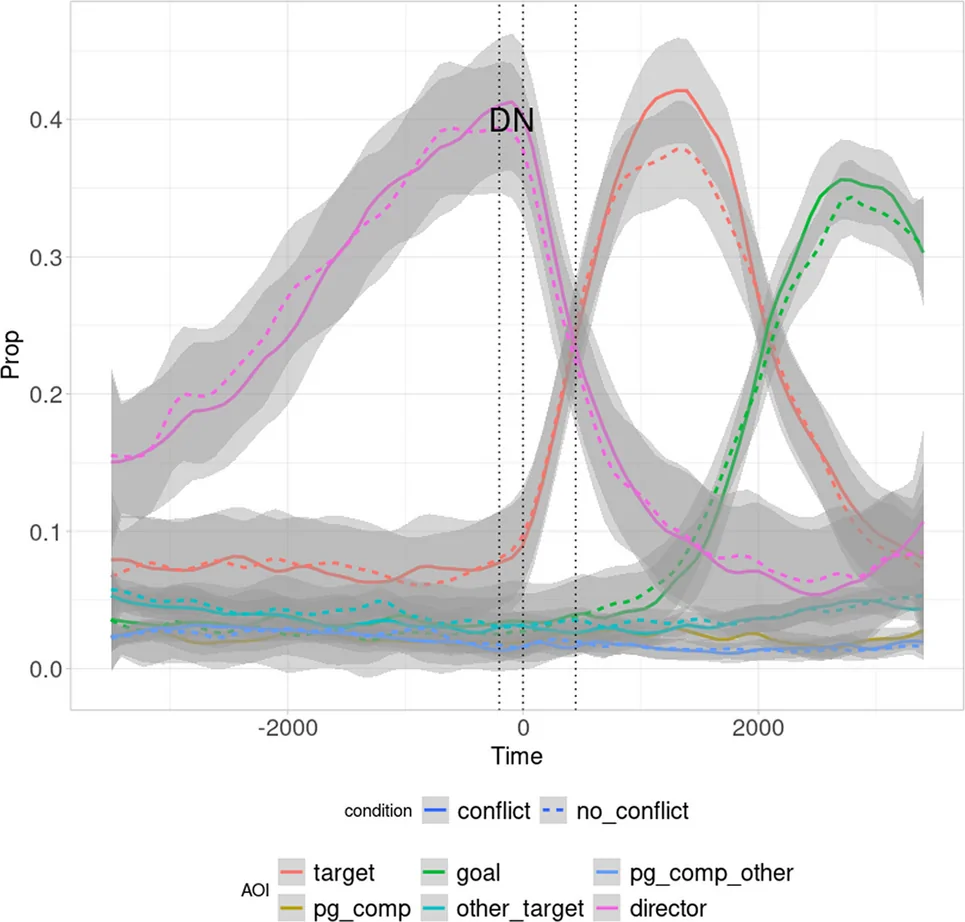
Gaze proportions including looks to the face of the director for a subset of participants (n=19). Dashed vertical lines mark determiner onset (D), noun onset (N) and noun offset (rightmost vertical line)

The multimodal nature of the dyadic interaction offers further points of departure for the investigation. Taking the directors’ gaze and prosody into account would provide further cues for data interpretation. This is, for instance, already evident by the fact that language-related ERP effects already differ between conditions before noun onset, even though the noun phrases used in the instructions are ambiguous until the noun. Joint attention with the director (cf. Egurtzegi et al., 2022; Sauppe, 2016, 2017) and prosodic patterns are plausible candidate cues, but a systematic analysis is beyond the scope of the present proof-of-concept paper. Our current data offer a first indication: although we were only able to track the face of the director in a subset of participants and trials (offering only a tentative analysis of these gazes), we observed that, on average, participants followed a very clear gazing strategy: they first gazed at the director’s face, then at the target object, and finally at the goal (Fig. 17).

We want to note that the deconvolution framework we use here is not restricted to time-domain voltage signals; as discussed by Ehinger and Dimigen (2019), the same regression and overlap-correction approach generalizes to any time-continuous EEG-derived signal, including band-limited power time courses. Oscillatory analyses (cf. Stinkeste et al., 2023) on the present and future datasets are therefore methodologically straightforward to integrate alongside their own preprocessing considerations.

Overall, the present study demonstrates that it is possible to collect EEG and eye-tracking data simultaneously in interactive, real-world settings. Our study shows the potential of such real-world interactions with multimodal data recordings: they enable us to study the coordination of multimodal processes, such as gaze control, language comprehension and production, as well as discourse and social phenomena during natural interaction situations. Future research must not only clarify the details of this interplay, but also establish new standards for preprocessing, analysis, and interpretation of such multimodal datasets through innovation, replication, and scientific exchange.

## Data Availability

Because of data protection rules, we cannot make the data publicly available, but the code for the analyses in this article is available at OSF (https://osf.io/5ds4z), along with two complete example data sets. Upon personal request, all data sets can be made available to other researchers.

## Code availability

All code used for this publication is publicly available online and actively maintained at OSF (https://osf.io/5ds4z) and at GitHub (https://github.com/XlinCLab/DGAME).
